# IP-to-MS: An Unbiased Workflow for Antigen Profiling

**DOI:** 10.1021/acs.jproteome.4c00837

**Published:** 2025-01-15

**Authors:** Stephanie Biedka, Svitlana Yablonska, Xi Peng, Duah Alkam, Mara Hartoyo, Hannah VanEvery, Daniel J. Kass, Stephanie D. Byrum, Kunhong Xiao, Yingze Zhang, Robyn T. Domsic, Robert Lafyatis, Dana P. Ascherman, Jonathan S. Minden

**Affiliations:** † Impact Proteomics, LLC., Pittsburgh, Pennsylvania 15206, United States; ‡ Center for Proteomics & Artificial Intelligence, Allegheny Health Network Cancer Institute, Pittsburgh, Pennsylvania 15205, United States; § Center for Clinical Mass Spectrometry, Allegheny Health Network Cancer Institute, Pittsburgh, Pennsylvania 15205, United States; ∥ Department of Biochemistry and Molecular Biology, University of Arkansas for Medical Sciences, Little Rock, Arkansas 72205, United States; ⊥ University of Pittsburgh School of Medicine, Pittsburgh, Pennsylvania 15261, United States; # Division of Pulmonary and Critical Care Medicine, Department of Medicine, University of Pittsburgh School of Medicine, Pittsburgh, Pennsylvania 15261, United States; ¶ Arkansas Children’s Research Institute, Little Rock, Arkansas 72202, United States; ∇ Department of Biomedical Informatics, University of Arkansas for Medical Sciences, Little Rock, Arkansas 72205, United States; ○ Division of Rheumatology and Clinical Immunology, Department of Medicine, University of Pittsburgh School of Medicine, Pittsburgh, Pennsylvania 15261, United States

**Keywords:** unbiased antigen identification, immunoprecipitation, mass spectrometry, autoimmune disease

## Abstract

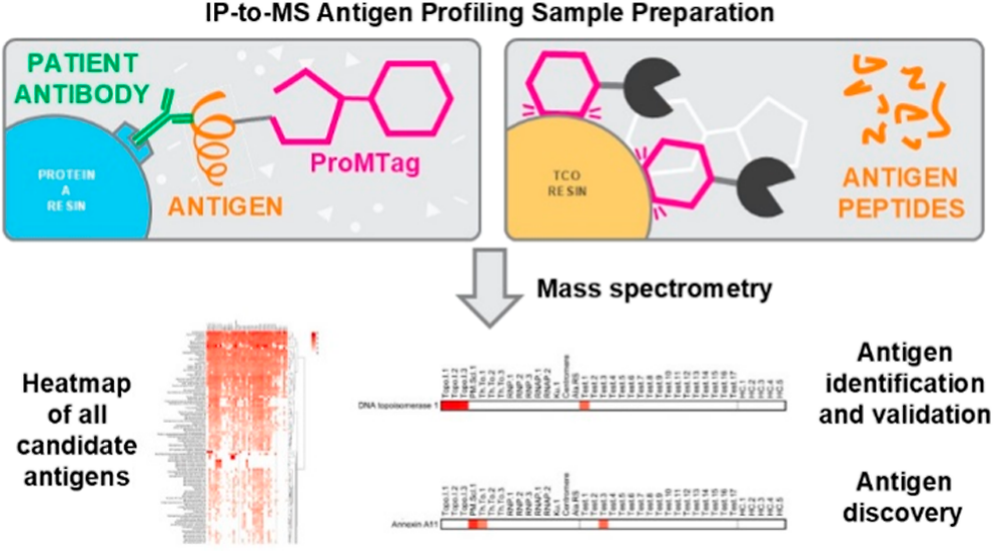

Immunoprecipitation is among the most widely utilized methods in biomedical research, with applications that include the identification of antibody targets and associated proteins. The path to identifying these targets is not straightforward, however, and often requires the use of chemical cross-linking and/or gel electrophoresis to separate targets from an overabundance of immunoglobulin protein. Such experiments are labor intensive and often yield long lists of candidate antibody targets. Here, we describe an unbiased immunoprecipitation-to-mass spectrometry (IP-to-MS) method that relies on a novel protein tag to separate low abundance immunoprecipitated proteins from overwhelmingly abundant immunoglobulins. We demonstrate that the IP-to-MS serotyping workflow is highly reproducible and can be used for the identification of novel, patient-specific antigen targets in multiple disease states. Furthermore, we show that IP-to-MS may outperform conventional methods of antibody detection, including enzyme-linked immunosorbent assay, while also enabling patient stratification beyond what is possible with traditional approaches.

## Introduction

Immunoprecipitation (IP) is one of the most widely used methods in biological and medical research.^1^ The breadth of IP applications is staggering, ranging from demonstration of cell/tissue-specific antigens to identifying associated coimmunoprecipitates, and from detection of viral antigens in patient samples to characterization of disease-specific autoantibodies in organ-based versus systemic autoimmune disease. Traditionally, the primary end point of a conventional IP assay has been gel electrophoresis to visualize the presence of the antigen and associated proteins.

Mass spectrometry (MS) has become vital for identifying target antigens and associated proteins in the above-mentioned applications.^2−5^ However, the presence of overwhelming levels of immunoglobulins presents a challenge for MS of immunoprecipitates. To facilitate MS of immunoprecipitated proteins, antibodies are either bound to protein A/protein G resin, or covalently cross-linked to bead-based matrices.^6,7^ The bound target and associated proteins, plus nonspecifically bound proteins and leached immunoglobulins, are eluted from the resin and separated by gel electrophoresis. Bands of interest are manually excised from the gel and processed by in-gel trypsin digestion. The resultant peptides are typically cleaned up by tip-based solid support columns and analyzed by liquid chromatography (LC)–MS. This is a complex, multistep process that requires several days of processing, significant amounts of input antibody, and advanced technical expertise.

Current IP–MS methods are plagued by immunoglobulin contamination and high background noise due to nonspecifically bound proteins. While cross-linking of antibodies to bead-based matrices reduces immunoglobulin contamination, these treatments require optimization to balance adequate antibody coupling with potential antibody inactivation caused by excess cross-linking reagent. The final outputs of these IP–MS experiments are relatively long lists of candidate proteins that require further study to differentiate between true target candidates and nonspecifically bound proteins.^8,9^

Patient-specific autoantibody/autoantigen discovery is among the most challenging applications of IP. Experiments designed for this purpose require patient serum or plasma (which is often in limited supply) and different amounts of tissue or cells to generate substrate antigens. The typical path to identifying new autoantigens begins with binding of patient antibodies to protein A resin and IP of radiolabeled protein from cell lysates.^10−13^ Radiolabeling of target proteins allows one to discriminate immunoprecipitated target proteins from immunoglobulin protein when separated by gel electrophoresis and visualized by autoradiography. Because radioactive proteins cannot be safely analyzed by MS, the autoradiograph is overlaid on a gel containing preparative amounts of immunoprecipitate. The matching bands are excised from the preparative IP gel, in-gel digested with trypsin, and subsequently analyzed by LC–MS. Serological proteome analysis is a similar approach which has been used to identify antigens in various diseases.^14−16^ While these methods are proven, they are labor intensive and technically demanding processes that cannot practically be applied to large numbers of patients.

As a result, current approaches for patient-specific autoantibody/autoantigen identification rely on a relatively small set of common autoantigens that can be used as standards for assessing targets of patient antibodies. Methods to detect relevant autoantibodies include enzyme-linked immunosorbent assay (ELISA), Ouchterlony double immunodiffusion (DID), and IP of radioactively tagged target proteins. These are all targeted assays that rely on previously identified autoantigens characterized by large-scale studies of common autoimmune diseases. Thus, they do not afford the opportunity to identify novel autoantibody specificities in rare autoimmune diseases lacking defined molecular characterization.

Protein microarrays provide another method to identify autoantibodies.^17^ While these allow for multiplex searches and offer a wider variety of potential autoantigen targets, they have certain shortcomings. First, they are biased in the number of potential autoantigen targets. Second, since these proteins are typically produced by microorganisms or in vitro, they do not carry the same post-translational modifications seen in human cells, their folded state is not certain, and their relative abundances do not match that of human cells, all of which impact antibody binding affinity. Thus, a truly unbiased population of putative autoantigens should come from human cells or tissues, with their typical folded structures, modifications, and abundances.^18^

In the context of autoimmune diseases that impact more than 20 million individuals in the US, roughly 30% do not have an identifiable autoantigen target using conventional methods of antibody detection.^19−21^ Therefore, there is a critical need for improved “agnostic” technological approaches that promote autoantigen discovery. To advance the methodology for identification and characterization of antigen/autoantigen targets, we have devised a novel, radiolabel-free method, referred to as IP-to-MS, that allows direct MS identification of antigens derived from any cell or tissue source that are recognized by antibodies found in the sera of individual patients. The method we describe here provides a means to isolate antigen targets that are largely free of immunoglobulins, allowing in situ digestion into tryptic peptides and subsequent MS analysis.

In this method, we have employed a protein capture reagent, ProMTag, to replace radiolabeling in separating immunoprecipitated antigens from immunoglobulins in IP eluates. The ProMTag is composed of three elements: a reversible protein-binding moiety that couples to amine groups on proteins in a pH-dependent manner; a flexible polyethylene glycol linker; and a methyltetrazine (MT) group for rapid, irreversible coupling to *trans*-cyclooctene (TCO), which is a well-known click chemistry pair.^22,23^

Although the ProMTag was originally developed for whole proteome capture and release,^24^ we have adapted this technology for unbiased IP of target autoantigens. This approach involving ProMTag demonstrates several critical features—namely, that binding of ProMTagged protein to TCO resin facilitates removal of the vast majority of serum immunoglobulins, and that ProMTagged proteins are amenable to release as intact proteins suitable for gel electrophoresis and/or MS identification. We demonstrate that the patient-specific IP-to-MS workflow allows for the rapid identification of novel autoantigens targeted by both previously characterized patient sera and sera previously designated as “seronegative” based on available clinical assays. Furthermore, we show that IP-to-MS can rival or surpass traditional methods of antibody detection, such as ELISA and Ouchterlony DID.

Because we are presenting a novel, unbiased approach to identifying putative autoantigens, we have devised slightly modified nomenclature to describe the output of this methodological approach. Rather than disease-specific antibodies, we refer to the output as patient-specific antigens. This designation indicates that the analysis is directed to a single individual, as opposed to a particular disease or patient population. Importantly, we do not use patient-matched lysates as the antigen sources for the experiments here; therefore, in these IP-to-MS assays the autoantibodies, but not the autoantigens, are patient-specific. Because the IP-to-MS method produces lists of putative target proteins, rather than antibodies per-se, we will refer to the output of the IP-to-MS method as potential autoantigens. The goal of employing IP-to-MS in this test case of analyzing sera from patients with autoimmune disease is to enable large-scale, quantitative data collection and analysis that will advance autoimmune disease classification.

## Experimental Procedures

### Cell Lysate and Sera Sources

K562 cells were grown by Cell Culture Company. We used the erythroleukemoid cell line K562 because these cells grow rapidly in culture and, although not tissue-specific, express a wide range of proteins that include commonly targeted autoantigens. As such, these cells represent the gold standard in the field of IP/autoantibody detection.

To prepare K562 cell lysates, cells were suspended in IP lysis buffer (IP-LB; Impact Proteomics, LLC.). The cells were sonicated on ice for 30–40 pulses at 30% power and 30% duty cycle on a Branson 450 Sonifier. The sample was centrifuged at 14,000 rpm for 20 min in a refrigerated benchtop centrifuge at 4 °C. The supernatant was removed, and the lysate was stored in 500 μL aliquots at −80 °C. Protein concentration was determined via a Pierce BCA assay (Thermo Fisher Scientific).

Patient-derived samples utilized in experiments shown in Figures 2–4 were collected as part of two separate longitudinal registries at the University of Pittsburgh encompassed by IRB-approved protocols 20030223 and 19090054. All patients (excluding healthy controls) had interstitial lung disease associated with an underlying connective tissue disease such as scleroderma, idiopathic inflammatory myopathy, or systemic lupus erythematosus.

### ProMTag Titration to Assess Protein Capture and Release

Six samples of 50 μg of K562 lysate were brought to a concentration of 0.5 mg/mL with IP-LB. Varying amounts of 30 mg/mL ProMTag was added (0, 0.25, 0.5, 1.0, 3.0, or 5.0 μL); 100% acetonitrile (ACN) (5.0, 4.75, 4.5, 4.0, 2.0, or 0 μL) was added to bring each sample to a final volume of 55 μL. Samples were incubated on ice for 30 min, then 10 μL of Quencher (Impact Proteomics, LLC.) was added to each sample to quench the labeling reaction. The samples were incubated on ice for another 30 min. Samples were then incubated at 4 °C with rotation for 2 h.

After the 2 h incubation was complete, 20% sodium dodecyl sulfate (SDS) (Bio-Rad) was added to a final concentration of 1.07% and the samples were incubated at room temperature with rotation for 15 min. During this incubation, 100 μL TCO agarose resin (Vector Laboratories, Inc.) per sample was washed once with water in resin capture (RC) tubes, which have a small slit in the bottom that allows the passing of liquid with minimal dead volume, but retains the solid resin (Impact Proteomics, LLC.). The samples were added to the TCO resin and incubated at room temperature with rotation for 15 min.

The flowthrough was collected by brief centrifugation of the RC tubes. The resin was then washed twice with 200 μL 100 mM (4-(2-hydroxyethyl)-1-piperazineethanesulfonic acid) (HEPES), 1% SDS and once with 200 μL 1 mM HEPES, 1% SDS. Each wash was collected in separate 1.5 mL tubes.

To elute the protein from the TCO resin, 75 μL of 100 mM formic acid (FA), 1% SDS was added followed by a 10 min incubation at room temperature with rotation. The elution step was repeated once, with both eluates being collected into the same 1.5 mL tube by brief centrifugation of the RC tubes.

For each sample, the flowthrough was combined with the first wash. The flowthrough + wash 1 fraction and the eluate were dried fully in a SpeedVac. The flowthrough + wash 1 fraction was resuspended in 100 μL water, and the eluate was resuspended in 100 μL 100 mM HEPES pH 8.0. The samples were run on 4–20% SDS-polyacrylamide gel electrophoresis (PAGE) gels (Bio-Rad) for 1.5 h at 120 V and the gels were stained with Coomassie Brilliant Blue R-250 (Bio-Rad). Quantification of the SDS-PAGE gels was done with ImageJ, with the blank region between the third and fourth lanes used for background subtraction. The percent of protein remaining in the flowthrough + wash 1 or the percent of protein in the eluate was quantified according to the pixel intensity of the gel images using ImageJ. The protein amount was calculated as a percentage of the “0 ProMTag” control for the “flowthrough + wash 1” gel or as a percentage of the 12.5 μg ProMTag per μg lysate sample for the “eluate” gel.

### Removal of Immunoglobulin before and after TCO Resin Elution

HeLa lysate in IP-LB was labeled with ProMTag for 30 min at room temperature at a ratio of 1.67 μg of protein per microgram of ProMTag. The labeling reaction was quenched by addition of Quencher followed by a 30 min incubation at room temperature.

During labeling of HeLa lysate, protein A resin was prepared as follows: 5 μL HiTrap MabSelect PrismA resin (Cytiva Life Sciences) was added to a RC tube and washed 3 times with 300 μL IPP buffer (Impact Proteomics, LLC.). The RC tube was sealed and the PrismA resin was resuspended in 25 μL IPP, then 2.5 μL polyclonal anti-Hsp90 (Abcam) and 10 μL patient serum with anti-Topo I autoantibody was added. Antibody binding to the PrismA resin was carried out for 1.5 h at room temperature with end-over-end rotation. The resin was then washed 4 times with 300 μL phosphate-buffered saline (PBS) and 3 times with 300 μL IPP. The resin was suspended in 50 μL IPP, then CyDye Cy5-NHS minimal dye (Cytiva) was added to a concentration of 20 μM and labeling was carried out for 15 min at room temperature. The labeling reaction was quenched by addition of Quencher followed by a 15 min incubation at room temperature. The resin was washed 2 times with 300 μL IPP and the RC tube was sealed with a stopper that prevents liquid flow.

The ProMTagged HeLa lysate (250 μL) was then added to the prepared PrismA resin. The resin was incubated with the lysate for 2 h at room temperature. The resin was then washed 4 times with 300 μL IPP. To elute proteins from the PrismA resin, 50 μL 100 mM HEPES pH 8.0, 1% SDS was added to the resin followed by a 10 min incubation at room temperature. This elution step was repeated once with both eluates for each sample being collected in the same 1.5 mL tube.

One-third of the PrismA resin eluate (33 μL) was used as the Load sample. This fraction, which contained Cy5-labeled proteins that had been bound and eluted from the PrismA beads (mostly Cy5-immunoglobulins), was labeled with CyDye Cy3-NHS at 20 μM concentration for 15 min at room temperature and then quenched by adding quencher followed by a 15 min incubation at room temperature. The second third of the PrismA eluate was added to 20 μL TCO resin that had been washed twice with 300 μL IP-WB3 in a RC tube. The TCO resin was incubated at room temperature for 30 min. The protein fraction that did not bind to the TCO beads was saved as the flowthrough fraction. The flowthrough fraction, which also contained Cy5-labeled proteins that had been bound and eluted from the PrismA beads (mostly Cy5-immunoglobulins), was labeled with CyDye Cy3-NHS at 20 μM concentration for 15 min at room temperature and quenched by the addition of quencher, as described above.

The final third of the PrismA eluate was added to 20 μL TCO resin that had been washed twice with 300 μL IP-WB3 in a RC tube. The TCO resin was washed 3 times with 300 μL 100 mM HEPES pH 8.0, 1% SDS, 10 mM dl-dithiothreitol (DTT); after the first addition of this buffer only, the sample was incubated with mixing for 30 min at room temperature. The TCO resin was then washed an additional 2 times with 300 μL 100 mM HEPES pH 8.0, then resuspended in 50 μL 100 mM HEPES pH 8.0. Protein bound to the TCO resin was labeled with CyDye Cy3-NHS minimal dye (20 μM) for 15 min at room temperature before being quenched by addition of quencher followed by a 15 min incubation at room temperature. The TCO resin was washed once with 300 μL 100 mM HEPES pH 8.0, then once with 300 μL 1% SDS. To elute protein from the TCO resin, 33 μL 100 mM FA, 1% SDS was added followed by a 30 min incubation at 37 °C. The Eluate fraction was collected and neutralized by addition of 1 M tetraethylammonium bromide (TEAB).

Samples were prepared for SDS-PAGE as follows: 33 μL of the load, flowthrough, and eluate fractions were mixed with 11 μL 4× sample buffer (Bio-Rad). Samples were run on a 4–20% SDS-PAGE gel (Bio-Rad) for ∼1.5 h at 120 V until the dye front had completely run out of the gel. Fluorescence images were acquired using a custom-built imager.^25^

### IP-to-MS of 37 Patient Sera

**Figure 1:**
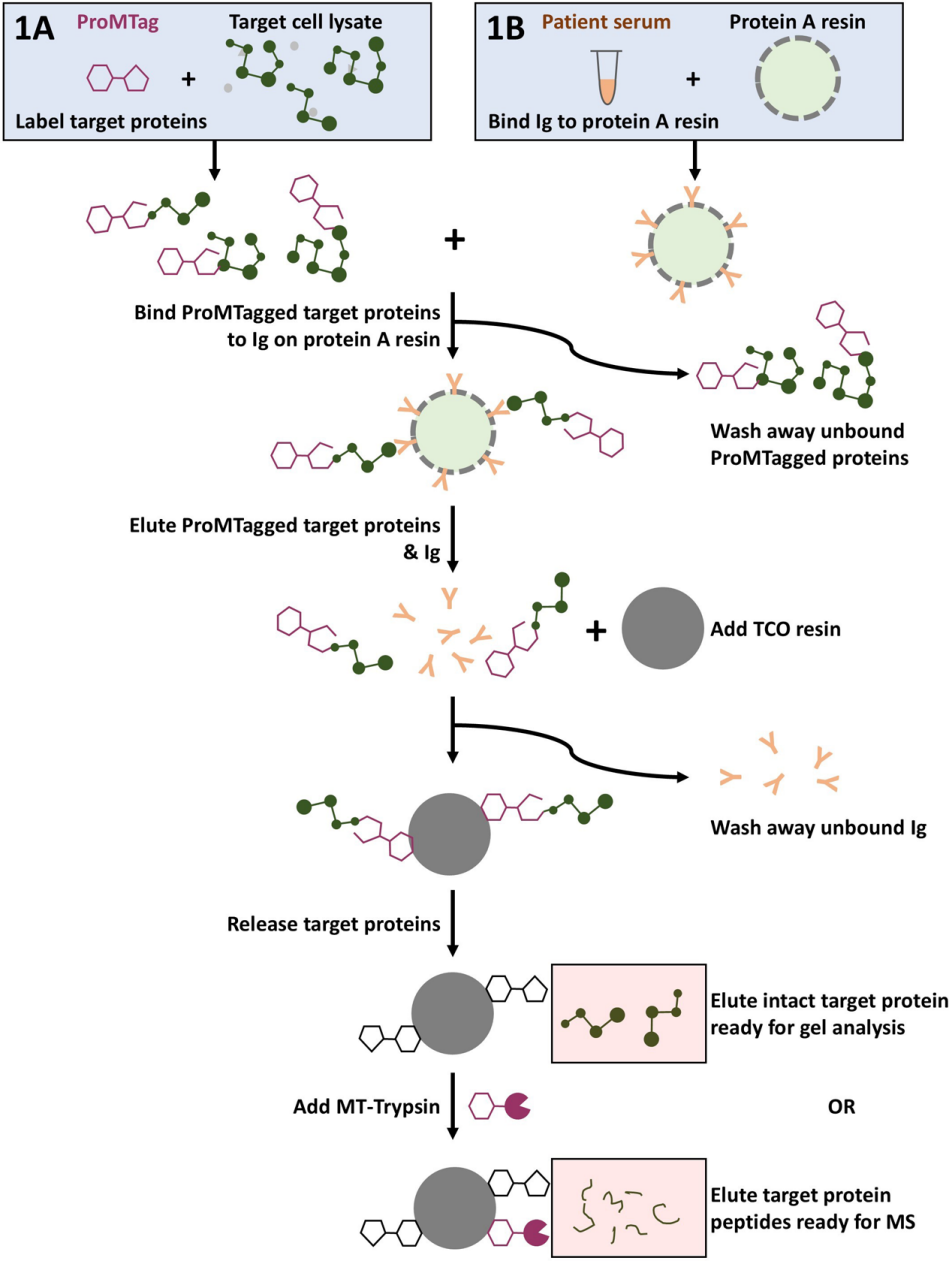
IP-to-MS workflow. The target cell lysate is labeled with ProMTag (A). Concurrently, antibodies, such as from a patient serum, are bound to protein A resin (B). The ProMTagged cell lysate is then added to the protein A resin and incubated at 4 °C for 2 h to bind the ProMTagged target proteins to the Ig on the protein A resin. Any unbound ProMTagged proteins are then washed away, and the ProMTagged target proteins and Ig are eluted from the protein A resin by a 15 min incubation in elution buffer. This IP eluate is added to TCO resin. The ProMTagged target proteins bind to the TCO resin, allowing the Ig and any remaining contaminants to be removed in a series of wash steps. The target proteins are released from the TCO resin in their original, unmodified state. Intact proteins can be eluted at this step, or MT-Trypsin can be added to digest the target proteins into MS-ready peptides.

Sera antibodies were bound to rProtein A Sepharose Fast Flow resin (Cytiva) (Figure 1, step 1B). Twenty microliters rProtein A resin was washed twice with 200 μL IPP buffer in RC tubes. The RC tubes were sealed, then 30 μL IPP buffer was added to the rProtein A resin. Next, 20 μL serum was added to each tube, followed by a 45 min incubation at 4 °C with rotation. After this incubation, the resin was washed four times with 300 μL IPP buffer and the RC tubes were sealed.

During binding of antibodies to rProtein A resin, K562 lysate was brought to a concentration of 0.5 mg/mL in IP-LB. ProMTag was added to the lysate at a ratio of 1.8 μg of ProMTag per microgram of protein, and the labeling reaction was incubated for 30 min on ice (Figure 1, step 1A). The labeling reaction was quenched by addition of 20 μL quencher per 200 μg lysate followed by a 30 min incubation on ice.

The ProMTagged K562 lysate (200 μg per sample) was then added to the prepared rProtein A resin (Figure 1, “Bind ProMTagged target proteins to Ig on protein A resin”). The samples were incubated at 4 °C for 2 h with rotation. The resin was then washed four times with 300 μL IPP buffer (Figure 1, “wash away unbound ProMTagged proteins”). To elute proteins from the rProtein A resin, 25 μL IP-elution buffer (IP-EB; Impact Proteomics, LLC.) was added to the resin followed by a 10 min incubation at room temperature with rotation (Figure 1, “elute ProMTagged target proteins & Ig”). This elution step was repeated once with both eluates for each sample being collected in the same 1.5 mL tube.

The eluates from the rProtein A resin were then added to 20 μL TCO resin that had been washed with 300 μL IP-wash buffer 3 (IP-WB3; Impact Proteomics, LLC.) in an RC tube (Figure 1, “add TCO resin”). The samples were incubated for 15 min at room temperature with rotation. The TCO resin was then washed as follows: three times with 300 μL IP-EB, three times with 300 μL IP-Wash Buffer 1 (IP-WB1; Impact Proteomics, LLC.), two times with 300 μL IP-Wash Buffer 2 (IP-WB2; Impact Proteomics, LLC.), one time with 300 μL IP-WB3, and two times with 300 μL ultrapure water (Figure 1, “wash away unbound Ig”).

Next, 40 μL 100 mM FA was added to the TCO resin (Figure 1, “release target proteins”) followed by 20 μL MT-Trypsin (Impact Proteomics, LLC.). Samples were incubated at 37 °C for 1 h. The eluate was collected by brief centrifugation of the RC tube, then an additional 40 μL 100 mM FA was added to the TCO resin. Samples were incubated at room temperature with rotation for 15 min. The RC tubes were once again briefly centrifuged into the same 1.5 mL tube as for the previous eluate (Figure 1, “elute target protein peptides ready for MS”). The eluates were then dried fully in a SpeedVac and stored at −80 °C.

### MS Analysis of Patient Autoantigens

Each sample was desalted using an Evotip Pure C18 disposable tip (EV2011, Evosep) following the manufacturer’s protocol with the following modifications: (1) for conditioning, instead of soaking the tips in 1-propanol, the Evotips were washed with 20 μL of 2-propanol (Optima LC/MS grade, Fisher Chemical) and centrifuged at 700*g* for 45–50 s until the solvent level was within 1 mm above the packing material; (2) the centrifugation steps for rinsing, equilibration, and washing were shortened to 10–15 s; (3) both the rinse and wash steps were repeated twice with 20 μL 0.1% FA in water (Optima LC/MS grade, Fisher Chemical).

With an Evosep One HPLC (Evosep), the desalted peptides were eluted off of the Evotip and loaded on to an Evosep EV1109 performance analytical column (8 cm × 150 μm inner diameter, 1.5 μm ReproSil Saphir C18 beads from Dr. Maisch). Peptide separation was carried out according to the manufacturer’s preset 11.5 min, 100 samples-per-day (SPD), method with 0.1% FA in water as solvent A and 0.1% FA in ACN as solvent B (Optima LC/MS grade, Fisher Chemical).

All mass spectrometric data were collected with a timsTOF Pro 2 mass spectrometer operated in the positive mode with TIMS enabled. A data-dependent acquisition with parallel accumulation-serial fragmentation (DDA-PASEF) method was utilized. Briefly, a full scan was first acquired for the mass range of *m*/*z* 100 to 1700, with the TIMS 1/*k*_0_ window set as 0.60–1.60 V·s/cm^2^. For 100% duty cycle (1.17 s cycle time) at a ramp rate of 9.42 Hz, the ramp time and accumulation time were set to 100 ms, respectively. The precursor isolation window was set to be linear across the *m*/*z* range, with a width of 2 *m*/*z* at 700 *m*/*z* and 3 *m*/*z* at 800 *m*/*z*. Precursors with charge states up to +5 that passed intensity threshold (2.5 × 10^3^) were then selected for fragmentation. Ten PASEF ramps were allowed during each cycle, with a dynamic exclusion duration of 0.4 min. Nitrogen was used as the collision gas, and the collision energy ranged from 20 to 59 eV across the defined TIMS 1/*k*_0_ window.

The MS data were searched with the Bruker Parallel Search Engine in Real-time (PaSER) platform (ver. 2023) against a reviewed human protein database from Uniprot (downloaded on 3/30/2023). Enzyme activity was set to be fully tryptic, with up to two missed cleavages allowed. The following variable modifications were considered: oxidation on M (+15.9949 Da) and phosphorylation on S/T/Y (+79.9663 Da), with up to two modification sites allowed for each peptide. Protein false discovery rate of 1% was applied. The mass tolerance for both the precursors and fragments were set to ±20 ppm, with each protein requiring at least one peptide identified within a mass error of ±10 ppm. Additional postsearch filters were applied to the peptides. XCorr score cutoff was set to 1.0 for peptides with a charge state of +1 and 0.8 for peptides with charge states of +2 to +4. DeltaCN cutoff was set to 0.1 for all peptides. A minimum percentage of identified by ions was set to 40%.

### Heatmap Generation

PaSER data in an Excel spreadsheet were filtered to remove immunoglobulin, keratin, and trypsin identifications. The spectral count values were converted to log_2_ values. These data were further filtered to remove any protein entry with log_2_ values less than 1 across all samples analyzed. The resultant spreadsheet was passed to R Studio and plotted using the Heatmap function and the ComplexHeatmap, circlize, and ggplot2 libraries.

### IP-to-MS Analysis of Four Characterized Patient Sera

Sera antibodies were bound to PrismA resin. Ten microliters PrismA resin was washed twice with 300 μL IPP buffer in RC tubes. The RC tubes were sealed, then 50 μL IPP buffer was added to the PrismA resin. Next, 20 μL serum was added to each tube, followed by a 45 min incubation at 4 °C with rotation. After this incubation, the resin was washed four times with 300 μL PBS, then two times with 200 μL IPP buffer, and the RC tubes were sealed.

During binding of antibodies to PrismA resin, K562 lysate was brought to a concentration of 1 mg/mL in IP-LB. Prior to optimization of ProMTag coupling, we used an irreversible version of the ProMTag, called PerMTag, which couples to proteins via an NHS ester. PerMTag was added to the lysate at a ratio of 1.8 μg of PerMTag per microgram of protein, and the labeling reaction was incubated for 30 min on ice. The labeling reaction was quenched by addition of 30 μL quencher per 200 μg lysate followed by a 30 min incubation on ice.

The PerMTagged K562 lysate (200 μg per sample) was then added to the prepared PrismA resin. The samples were incubated at 4 °C for 2 h with rotation. The resin was then washed four times with 300 μL IPP buffer. To elute proteins from the PrismA resin, 25 μL IP-EB was added to the resin followed by a 10 min incubation at room temperature with rotation. This elution step was repeated once with both eluates for each sample being collected in the same 1.5 mL tube.

The eluates from the PrismA resin were then added to 40 μL TCO resin that had been washed with 300 μL IP-WB3 in an RC tube. The samples were incubated for 15 min at room temperature with rotation. The TCO resin was then washed as follows: one time with 300 μL IP-WB1, two times with 300 μL IP-WB2, one time with 300 μL IP-WB3, and two times with 300 μL ultrapure water.

Next, 40 μL 100 mM FA was added to the TCO resin followed by 20 μL MT-Trypsin. Samples were incubated at 37 °C for 1 h. The eluate was collected by brief centrifugation of the RC tube, then an additional 40 μL 100 mM FA was added to the TCO resin. Samples were incubated at room temperature with rotation for 15 min. The RC tubes were once again briefly centrifuged into the same 1.5 mL tube as for the previous eluate. The eluates were then dried fully in a SpeedVac and stored at −80 °C.

Tryptic peptides were separated by reverse phase XSelect CSH C18 2.5 μm resin (Waters) on an in-line 150 × 0.075 mm column using an UltiMate 3000 RSLCnano system (Thermo Fisher Scientific). Peptides were eluted using a 60 min gradient from 98:2 to 65:35 buffer A/B ratio (buffer A = 0.1% FA, 0.5% ACN, buffer B = 0.1% FA, 99.9% ACN). Eluted peptides were ionized by electrospray (2.2 kV) followed by mass spectrometric analysis on an Orbitrap Exploris 480 mass spectrometer (Thermo Fisher Scientific). To assemble a chromatogram library, six gas-phase fractions were acquired on the Orbitrap Exploris with 4 *m*/*z* DIA spectra (4 *m*/*z* precursor isolation windows at 30,000 resolution, normalized AGC target 100%, maximum inject time 66 ms) using a staggered window pattern from narrow mass ranges using optimized window placements. Precursor spectra were acquired after each DIA duty cycle, spanning the *m*/*z* range of the gas-phase fraction (i.e., 496–602 *m*/*z*, 60,000 resolution, normalized AGC target 100%, maximum injection time 50 ms). For wide-window acquisitions, the Orbitrap Exploris was configured to acquire a precursor scan (385–1015 *m*/*z*, 60,000 resolution, normalized AGC target 100%, maximum injection time 50 ms) followed by 50 × 12 *m*/*z* DIA spectra (12 *m*/*z* precursor isolation windows at 15,000 resolution, normalized AGC target 100%, maximum injection time 33 ms) using a staggered window pattern with optimized window placements. Precursor spectra were acquired after each DIA duty cycle.

Following data acquisition, data were searched using an empirically corrected library and a quantitative analysis was performed to obtain a comprehensive proteomic profile. Proteins were identified and quantified using EncyclopeDIA^26^ and visualized with Scaffold DIA using 1% false discovery thresholds at both the protein and peptide level. Protein MS^2^ exclusive intensity values were assessed for quality using ProteiNorm, a tool for systematic evaluation of normalization methods, imputation of missing values and comparisons of multiple differential abundance methods.^27^ Normalization methods evaluated included log_2_ normalization (log_2_), median normalization (median), mean normalization (mean), variance stabilizing normalization (VSN),^28^ quantile normalization (quantile),^29^ cyclic loess normalization (cyclic loess),^30^ global robust linear regression normalization,^31^ and global intensity normalization (global intensity). The individual performance of each method was evaluated by comparing of the following metrices: total intensity, pooled intragroup coefficient of variation, pooled intragroup median absolute deviation, pooled intragroup estimate of variance, intragroup correlation, sample correlation heatmap (Pearson), and log_2_-ratio distributions. The VSN normalized data were used to perform statistical analysis using linear models for microarray data (limma) with empirical Bayes (eBayes) smoothing to the standard errors.^30^ Proteins with an FDR adjusted *p*-value < 0.05 and a fold change > 2 were considered significant.

### IP of Standard Set without ProMTag

Sera antibodies or anti-Hsp90 were bound to rProtein A resin. Twenty microliters rProtein A resin was washed twice with 200 μL IPP buffer in RC tubes. The RC tubes were sealed, then 30 μL IPP buffer was added to the rProtein A resin. Next, either 20 μL serum or 5 μL anti-Hsp90 was added to each tube, followed by a 45 min incubation at 4 °C with rotation. After this incubation, the resin was washed three times with 200 μL IPP buffer, then resuspended in 50 μL IPP buffer. Antibodies were labeled with CyDye Cy5-NHS minimal dye (Cytiva; 14 μM) for 15 min at 4 °C with rotation. The labeling reaction was quenched by addition of 2 μL quencher followed by a 30 min incubation at 4 °C with rotation. The resin was then washed three times with 200 μL IPP and the RC tubes were sealed.

During binding of antibodies to rProtein A resin, 1260 μg K562 lysate was brought to a concentration of 0.5 mg/mL in IP-LB. Proteins were labeled with CyDye Cy3-NHS minimal dye (Cytiva; 5.9 μM) for 30 min on ice. The labeling reaction was quenched by addition of Quencher followed by a 30 min incubation on ice.

The Cy3-K562 lysate (200 μg) was then added to the Cy5-antibody rProtein A resin. The samples were incubated at 4 °C for 2 h with rotation. The resin was then washed three times with 200 μL IPP buffer. To elute proteins from the rProtein A resin, 25 μL IP-EB was added to the resin followed by a 10 min incubation at room temperature with rotation. This elution step was repeated once with both eluates for each sample being collected in the same 1.5 mL tube.

The eluates were run on a 4–20% SDS-PAGE gel (Bio-Rad) for ∼1.5 h at 120 V until the dye front had completely run out of the gel. Fluorescence images were acquired using a FluorChem M imager (ProteinSimple). Images were cropped and despeckled using ImageJ.

### IP of Standard Set without ProMTag, with Cross-Linking

Binding of sera antibodies or anti-Hsp90 to rProtein A resin and labeling with Cy5-NHS was carried out as described above. The following additional cross-linking steps were carried out. Following the 30 min quenching incubation, the resin was washed once with 200 μL 200 mM triethanolamine (TEA) pH 8.9 (Sigma-Aldrich). Next, 100 μL 50 mM dimethyl pimelimidate (Sigma-Aldrich) in TEA was added and the samples were incubated at 4 °C for 1 h with mixing. The resin was washed once with 200 μL 200 mM TEA, then 100 μL 100 mM ethanolamine pH 8.9 (Sigma-Aldrich) was added followed by a 15 min incubation at 4 °C with rotation. The resin was washed once with 200 μL 100 mM FA and then three times with 200 μL IPP buffer.

Labeling of the K562 lysate, binding of the Cy3-K562 lysate to the cross-linked Cy5-antibody resin, washing of the rProtein A resin, and elution from the rProtein A resin were carried out as described above. Eluates were run on a 4–20% SDS-PAGE gel (Bio-Rad) for ∼1.5 h at 120 V until the dye front had completely run out of the gel. Fluorescence images were acquired using a FluorChem M imager. Images were cropped and despeckled using ImageJ.

### IP of Standard Set with ProMTag

Binding of sera antibodies or anti-Hsp90 to rProtein A resin and labeling with Cy5-NHS was carried out as described above. Labeling of the K562 lysate was carried out as described above with the following modification: immediately after adding the CyDye Cy3-NHS to the K562 lysate, ProMTag was added at a ratio of 1.8 μg of ProMTag per microgram of protein. The labeling reaction was incubated and quenched as described above. Binding of the Cy3-K562 lysate to the Cy5-antibody resin, washing of the rProtein A resin, and elution from the rProtein A resin were carried out as described above.

The eluates from the rProtein A resin were then added to 20 μL TCO resin that had been washed with 200 μL IP-WB3 in a RC tube. The samples were incubated for 15 min at room temperature with rotation. The TCO resin was then washed as follows: three times with 200 μL IP-EB, three times with 200 μL IP-WB1, two times with 200 μL IP-WB2, one time with 200 μL IP-WB3, and two times with 200 μL ultrapure water.

To elute proteins from the TCO resin, 22 μL 100 mM FA, 1% SDS was added to the resin followed by a 10 min incubation at room temperature with rotation. This elution step was repeated once with both eluates for each sample being collected in the same 1.5 mL tube. The eluate was neutralized by addition of 7 μL 1 M TEAB. Eluates were run on a 4–20% SDS-PAGE gel (Bio-Rad) for ∼1.5 h at 120 V until the dye front had completely run out of the gel. Fluorescence images were acquired using a FluorChem M imager. Images were cropped and despeckled using ImageJ.

### MS Identification of Bands Excised from Standard Sera Set Gels

The bands excised from the SDS-PAGE gels shown in Figure S3 were rinsed and equilibrated in 0.1 M ammonium bicarbonate. Proteins were reduced with DTT and alkylated with 2-iodoacetamide while in the gel. For efficient digestion of proteins, the gel bands were crushed with a pestle in tight-fitting 1.5 mL tubes, then the gel was dehydrated with ACN. MT-Trypsin in 0.1 M ammonium bicarbonate was added to the dehydrated gel, and proteins were digested for 2 h at 37 °C. Peptides were extracted from the gel using FA and ACN, and MT-Trypsin was removed from the solution via capture on TCO resin. The final peptide eluate was lyophilized prior to MS.

Twenty percent of yielded peptides were separated by an Evosep One high-performance liquid chromatography system (Evosep) and then subjected to tandem mass spectrometric analyses by a timsTOF Pro 2 mass spectrometer (Bruker). Briefly, each Evotip Pure desalting column (EV2011, Evosep) was conditioned and equilibrated following the protocol established by the manufacturer with minor changes. Decreased centrifugation times (10–15 s at 700*g*) were applied to keep the solvent level within 2 mm above the disks in the tip. Additional washing steps (2× 20 μL 0.1% FA in water) were performed after peptide loading. The Evosep 100 samples-per-day (100SPD) method was applied for peptide separation over an 11.5 min gradient using 0.1% FA in water and 0.1% FA in ACN (Optima LC/MS grade, Fisher Chemical) as solvent A and B respectively.

For data acquisition, a standard data-dependent acquisition with parallel accumulation-serial fragmentation (DDA-PASEF) method was utilized as described above in the “MS Analysis of Patient Autoantigens” section.

All MS data were searched with MaxQuant (ver. 2.1.3.0, Max Planck Institute of Biochemistry)^32^ against a reviewed Swiss-Prot human protein sequence database (downloaded on 01/22/2023). For peptide identification, the following modifications were considered: carbamidomethyl on cysteine (static), oxidation on methionine (variable), and acetylation of protein N-terminus (variable), with up to two variable modifications allowed for each peptide. Mass tolerance was set at ±10 pm, and both unique and razor peptides were used for protein quantification. Default score and delta score cutoffs were applied for peptide filtering, and 1% false discovery rate (FDR) was applied on both peptide-spectrum-match and protein levels.

### Cohort Development for Comparison of IP-to-MS to Conventional IP, Double Immunodiffusion, and ELISA

Sera derived from a subset of SSc patients from the University of Pittsburgh longitudinal cohort previously classified as ATA+ by IP were assessed by a combined ELISA and DID testing process using recombinant human topoisomerase I as substrate antigen and known ATA+ sera as reference samples. A subset of these sera samples was further analyzed by IP-to-MS.

### ELISA of SSc Patient Sera

IgG anti-full-length human topoisomerase I antibody levels were measured using standard solid-phase ELISA according to the following protocol. Ninety-six well microtiter plates (Thermo Fisher Scientific) were coated with baculovirus-expressed, full-length human topoisomerase I purified from Sf9 insect cells (1.0 μg/mL)^33^ versus no antigen in carbonate buffer (50 mM NaHCO_3_/Na_2_CO_3_, pH 9.6) and incubated overnight at 4 °C. The plates were washed three times with PBS containing 0.05% Tween-20. Wells were blocked with PBS-0.05% Tween-20 containing 1% BSA, then appropriately diluted serum samples (1:10,000) were added for 2 h. The plates were washed and then incubated for 60 min with horseradish peroxidase-conjugated goat antihuman IgG (1:10,000; Abcam, Massachusetts, USA). Enzymatic reactions were initiated using 3,3′,5,5′-tetramethyl-benzidine (TMB) (Sigma-Aldrich) and then terminated with 1 N H_2_SO_4_. Color development was measured at 450 nm with a BioTek Synergy 2 ELISA Reader (BioTek Company) and quantified as standard units following conversion of adjusted OD_450_ values [OD_450_ substrate antigen – OD_450_ no antigen] using a standard reference serum and dose response curve. All assays were performed in duplicate wells.

### Ouchterlony DID of SSc Patient Sera

Sixty mm Petri dishes were used to prepare 0.8% agarose gel [0.8% w/v ultrapure agarose (Thermo Fisher Scientific) diluted in PBS] templates for Ouchterlony DID assays. Individual wells were loaded with either 16 μL of recombinant topoisomerase antigen diluted in PBS to a final concentration of 0.0625–0.25 mg/mL or patient sera at various dilutions (undiluted, 1:4 dilution in PBS) and incubated at room temperature in a humidified chamber for 48–72 h. Precipitin lines were then visualized with white light and scored according to line intensity: 1-faint line with bright light, 2-moderately strong line with bright light, 3-faint line with ambient light, 4-moderate/strong line with ambient light.

### IP-to-MS of SSc Patient Sera

K562 lysate was brought to a concentration of 0.5 mg/mL in IP-LB. PerMTag was added to the lysate at a ratio of 3.33 μg of lysate per microgram of PerMTag. The labeling reaction was incubated on ice for 30 min, then it was quenched by addition of Quencher followed by a 30 min incubation on ice. The labeled lysate was split into four equal aliquots and stored at −80 °C.

To bind sera antibodies to rProtein A Sepharose Fast Flow resin, 10 μL resin was washed twice with 100 μL IPP buffer in RC tubes. The RC tubes were sealed, then 30 μL IPP buffer was added to suspend the resin. Next, 10 μL serum was added to each tube, followed by a 45 min incubation at 4 °C with rotation. During this incubation, the PerMTagged K562 lysate that had been stored at −80 °C was thawed on ice. Following the 45 min incubation, the rProtein A resin was washed four times with 100 μL IPP buffer, and the RC tubes were resealed.

The PerMTagged K562 lysate (100 μg per sample) was added to the prepared rProtein A resin and the samples were incubated at 4 °C for 2 h with rotation. The resin was washed four times with 100 μL IPP buffer. To elute proteins from the rProtein A resin, 12.5 μL IP elution buffer was added to the resin followed by a 10 min incubation at room temperature with rotation. This elution step was repeated once with both eluates for each sample being collected in the same 1.5 mL tube.

To the combined 25 μL IP eluates, 1 μL 100 mM DTT was added followed by a 30 min incubation at 56 °C. To alkylate the samples, 1 μL 200 mM IAA was added followed by a 30 min incubation at room temperature in the dark.

The reduced and alkylated IP eluates were then added to 10 μL TCO resin that had been washed with 100 μL IP-WB3. The samples were incubated for 15 min at room temperature with rotation. The TCO resin was then washed as follows: two times with 100 μL IP-EB, two times with 100 μL IP-WB1, two times with 100 μL IP-WB2, one time with 100 μL IP-WB3, and two times with 100 μL ultrapure water.

Next, 20 μL 100 mM FA was added to the TCO resin followed by 10 μL MT-Trypsin. Samples were incubated at 37 °C for 1 h, then the eluate was collected by brief centrifugation of the RC tube. An additional 20 μL 100 mM FA was added to the TCO resin and the samples were incubated at room temperature with rotation for 15 min. The RC tubes were briefly centrifuged into the same tube as for the previous eluate. Peptides were fully dried in a SpeedVac and stored at −80 °C.

MS of peptides was carried out as described above in the “MS Analysis of Patient Autoantigens” section with one modification: in addition to oxidation on M and phosphorylation on S/T/Y, carbamidomethyl on C was also considered. The heatmap of the SSc IP-to-MS results was carried out as described above in the “Heatmap Generation” section.

### Statistical Analysis of DID, ELISA, and IP-to-MS Results

Concordance of ELISA and DID results was assessed through contingency table analysis. Phenotypic differences between ATA+ and ATA– groups classified by the combined ELISA + DID method were analyzed through a combination of parametric and nonparametric statistical tests, assuming DID to be the gold standard method for ATA detection.

## Results

### The IP-to-MS Workflow

The IP-to-MS workflow is outlined in Figure 1. First, proteins from the desired cell or tissue source are labeled with ProMTag so that greater than 85% of the input protein is capturable by TCO resin. Concurrently, patient serum is exposed to protein A resin to capture immunoglobulins. After washing unbound proteins from the protein A resin, the ProMTagged whole-cell proteome (autoantigen target pool) is exposed to patient antibodies bound to the protein A resin. Unbound ProMTagged proteins are washed away, leaving bound ProMTagged target proteins and immunoglobulins bound to the protein A resin.

All proteins bound to the protein A resin are then released by SDS denaturation. This mixture of ProMTagged target proteins and untagged immunoglobulins is exposed to TCO resin, which covalently cross-links the ProMTagged target proteins. The TCO resin is then washed extensively to remove immunoglobulins and any remaining contaminants.

At this point in the workflow, intact target proteins are released from the TCO resin by a pH change which reverses the linkage between the ProMTag and the protein, leaving a chemically unaltered protein ready for further analysis either by gel electrophoresis or trypsin digestion and MS analysis. To enable rapid trypsin digestion, MT tagged trypsin (MT-Trypsin) is added in excess to digest the target proteins within 1 h. MT-Trypsin is removed from solution by covalent coupling to the TCO resin, thus yielding tryptic peptides ready for MS analysis.

A set of control experiments were performed to test for ProMTag protein capture and release under IP conditions. More than 85% of the input protein target pool was captured and released (Figure S1A). We further demonstrated that the vast majority of untagged antibodies were separated from ProMTagged target proteins, thus enabling direct IP of target proteins and efficient antibody removal (Figure S1B,C).

### IP-to-MS Analysis of Individuals with Interstitial Lung Disease

**Figure 2:**
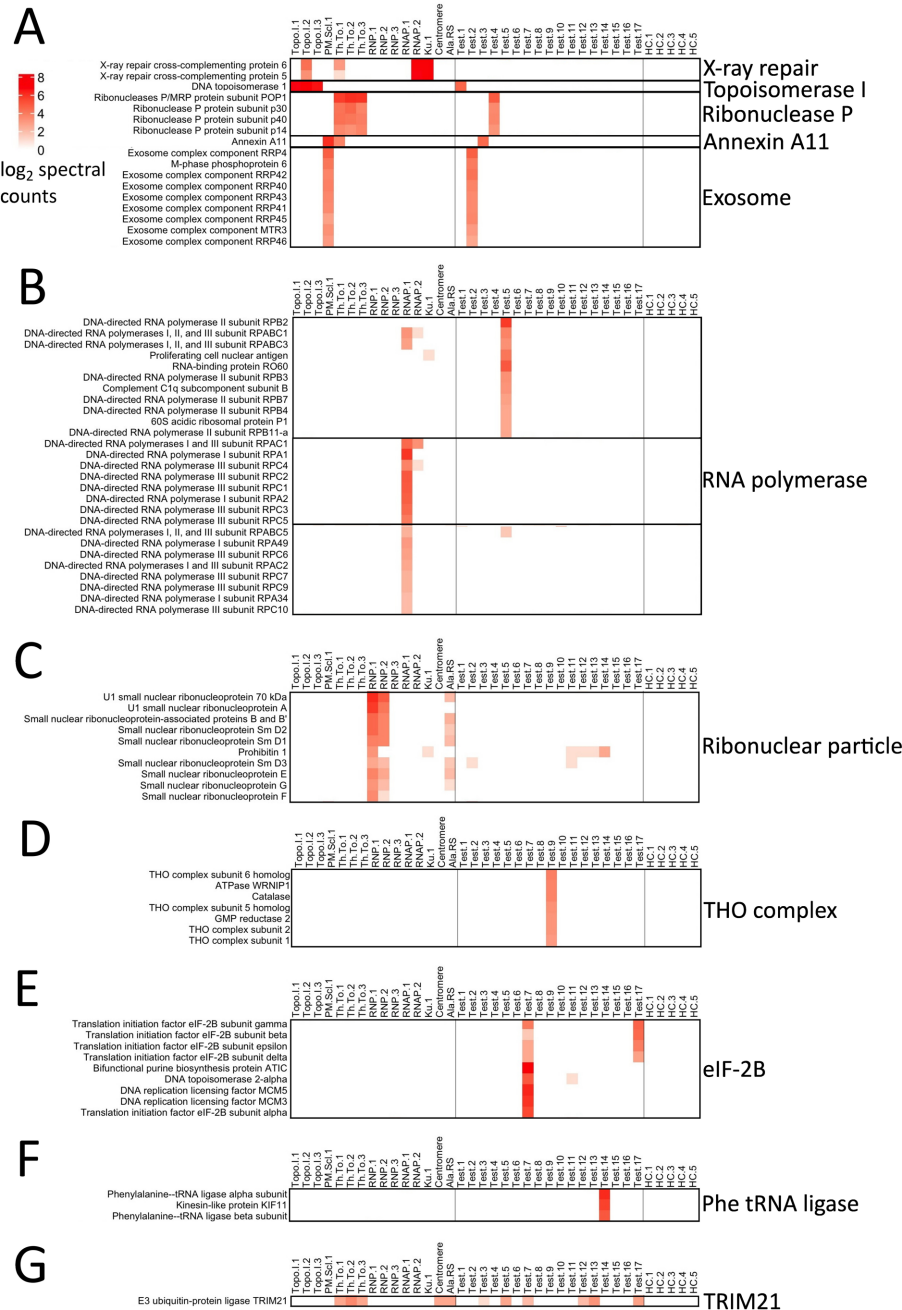
IP-to-MS of 37 patient sera against K562 lysate. IP-to-MS of 37 patient sera were carried out against a ProMTagged K562 lysate. These samples were broken into three groups. The first set of 15 samples (Topo.I.1 through Ala.RS) were prepared with patient sera with known autoantigens. The next set of 17 samples (Test.1 through Test.17) were prepared with uncharacterized patient sera. The remaining 5 samples (HC.1 through HC.5) were prepared with healthy control sera. MS data were filtered to remove immunoglobulin, keratin, and trypsin, and the data were displayed as a heatmap where the log_2_ spectral counts were plotted. Select areas of the heatmap with protein complexes of interest are shown (A–G). The full heatmap is shown in Figure S2.

To demonstrate the utility of the IP-to-MS workflow, we assessed 32 sera from patients diagnosed with connective tissue disease-associated interstitial lung disease (CTD-ILD), and five sera from healthy individuals (Figures 2 and S2; Table S1). Fifteen of the CTD-ILD sera were previously characterized and known to recognize proteins including topoisomerase I, exosome complex, RNase P complex, ribonuclear protein (RNP) complex, RNA polymerase, X-ray repair complex, centromere, and alanine aminoacyl tRNA synthetase. These were used as positive controls. The remaining 17 CTD-ILD patient serum samples had not been fully characterized and therefore had autoantibodies of unknown specificity. All of these samples were processed by the IP-to-MS workflow. MS analysis was performed on a Bruker timsTOF Pro2 instrument using an 11.5 min gradient routine. Because the IP-to-MS samples were known to have relatively low complexity, the shorter MS run time allowed for higher throughput without sacrificing sensitivity. The data were filtered to remove immunoglobulin, keratin, and trypsin identifications. The data were then displayed as a heatmap where the log_2_ spectral counts were plotted. Proteins with at least two spectral counts in at least one sample were included in the heatmap.

The top region of the heatmap contains proteins that were detected in most or all of the samples, ranging from high spectral counts to low spectral counts (Figure S2). Because of the broad distribution across the majority of sera, these proteins were deemed to be nonspecific binding proteins. Several of these proteins were seen only in the patient sera, and not in the healthy controls, suggesting more specific recognition by cognate autoantibodies.

Below this region of putative nonspecific binding proteins lies proteins that are clearly sample-specific. Vertical streaks are an obvious feature of this lower section of the heatmap. These groups of proteins that were specifically immunoprecipitated by individual patient sera often correspond to subunits of particular protein complexes, such as ribonuclease P, exosome, RNA polymerases, and RNP complexes (Figure 2A–C). Because the ProMTag is added under native conditions, the IP-to-MS method immunoprecipitates protein complexes.

With respect to the previously characterized sera that were used as positive controls, the IP-to-MS method positively identified expected proteins in 14 out of 15 samples. For example, the three known sera with topoisomerase I reactivity (Topo.I.1 through Topo.I.3) detected this protein in the IP-to-MS workflow (Figure 2A). In addition, topoisomerase I was also detected in one of the uncharacterized patient serum samples (Test.1). The Ku.1 patient serum containing X-ray repair cross-complementing proteins 5 and 6 antibodies immunoprecipitated corresponding antigen targets in the IP-to-MS workflow. These proteins were also identified in the Topo.I.2, Th.To.1, and RNAP.2 patient sera, which were also reactive against topoisomerase I, ribonuclease P and annexin 11, and RNA polymerases, respectively (Figure 2A,B).

All 17 test samples reacted to specific sets of proteins. Many of the test samples targeted proteins that were also recognized by some of the sera from the positive control group, including topoisomerase I (Test.1), ribonuclease P subunits (Test.4), exosome subunits (Test.2), and RNA polymerase subunits (Test.5), among others (Figure 2A,B). However, the remaining Test samples appeared to contain antibodies to previously uncharacterized autoantigen proteins. Of note is annexin A11, which was immunoprecipitated by Test.3 serum, as well as by positive control sera that had reactivity to the exosome and ribonuclease P (Figure 2A). Other interesting autoantigens were observed, including the THO complex (Figure 2D, Test.9), eukaryotic translation initiation factor 2B (eIF-2B) (Figure 2E, Test.7 and Test.17), and phenylalanine tRNA ligase (Figure 2F, Test.14). Several proteins were immunoprecipitated by many of the patient sera, but not by the healthy control sera. For example, 11 of the 32 patient sera immunoprecipitated Ro52/TRIM21 (tripartite motif-containing protein 21) (Figure 2G), a known autoantigen in Sjögren’s syndrome, systemic lupus erythematosus, and other rheumatic autoimmune diseases.^34−39^

This test of the IP-to-MS workflow demonstrated that IP-to-MS can correctly identify autoantigen targets of previously characterized sera from patients with autoimmune disease. It also showed that all test sera contained autoantibodies to previously known autoantigens and/or novel autoantigens, demonstrating the potential utility of this method for discovering autoantigen targets recognized by patient sera.

### IP-to-MS Reproducibility

**Figure 3:**
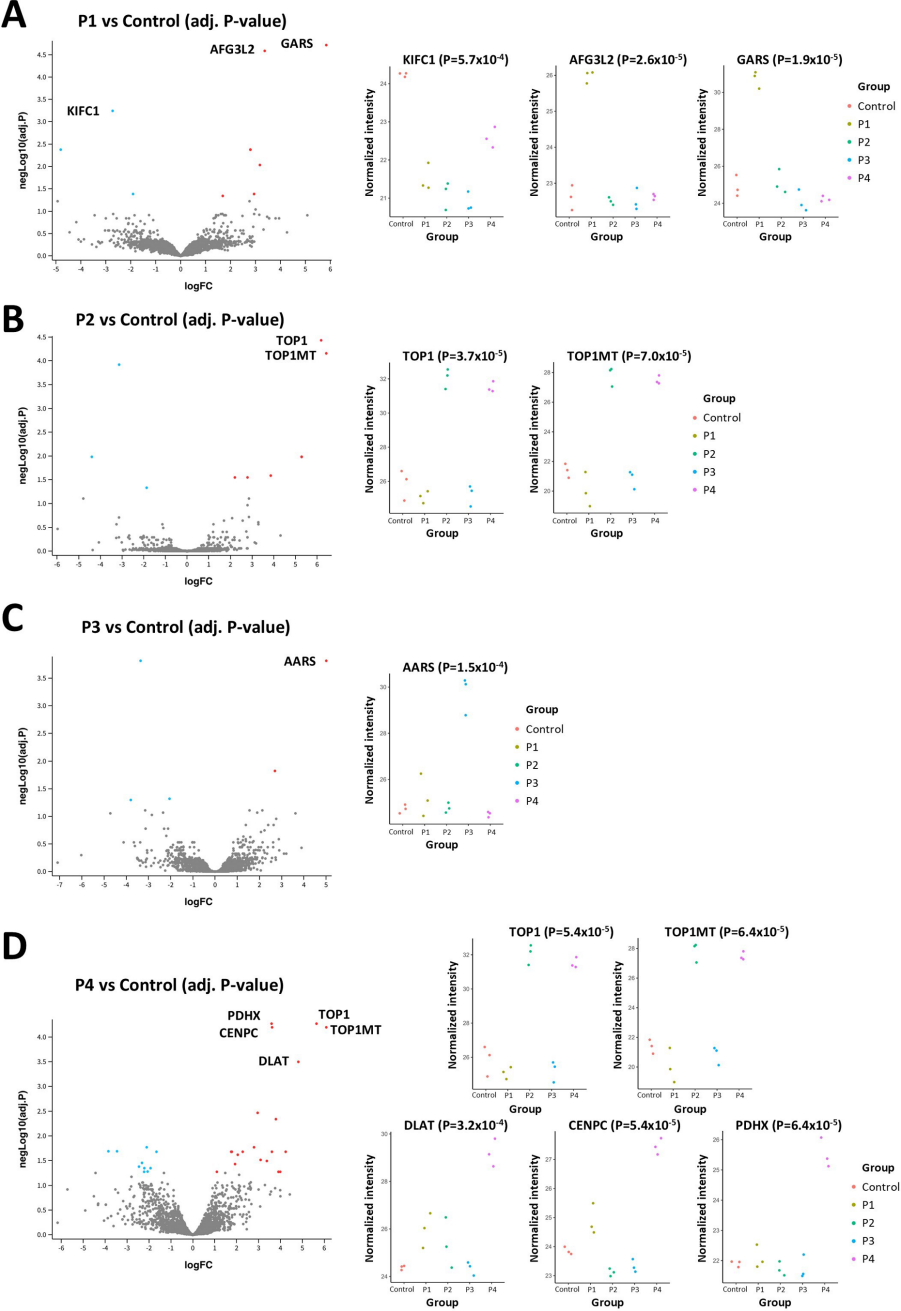
IP-to-MS of four characterized patient sera against K562 lysate. IPs of four patient sera with known autoantigens were carried out against a ProMTagged K562 lysate and compared to an IP of serum from a healthy control patient. Immunoprecipitated antigens were identified by MS. The volcano plots compare each patient serum to control serum where the average log fold change (FC) was plotted against the average −log_10_ probability (*P*) score for proteins commonly detected across samples. Only proteins with log_2_ FC > ± 1 and −log_10_ FDR adjusted *P*-value > 1.3 were considered. The normalized intensity of specific autoantigens found in each sample are plotted to the right of the volcano plots. The P1 and P3 sera were obtained from patients with myositis (A,C), and the P2 and P4 sera were taken from patients with scleroderma (B,D). The FDR adjusted *P*-value for each protein is shown in parentheses.

To demonstrate the reproducibility of the IP-to-MS workflow, four patient sera with known autoantibodies and a healthy control serum were assessed in triplicate. Each IP-to-MS sample was analyzed by a 1 h gradient on an Orbitrap Exploris 480 MS. Figure 3 shows volcano plots comparing each patient serum to control serum taken from a healthy individual where the average log_2_ fold change (log FC) is plotted against the average −log_10_ FDR adjusted probability (neglog(adj. P)) score for proteins commonly detected across samples. We took a very stringent approach and focused on proteins with log_2_ FC > ± 1 and −log_10_ adj. *P* > 1.3. Reproducibility was quantified using the Adjusted *P*-value which is calculated from the confidence values, standard error, and corrected for the False Discovery Rate according to Benjamini and Hochberg.^40^ These data are contained in the Table S6 excel spreadsheet, where a total of 2676 proteins were identified across all 15 samples.

Patients 1 and 3 (P1 and P3) have myositis, which is charactered by presence of antiaminoacyl tRNA synthetase antibodies.^41^ P1 serum contained antibodies that immunoprecipitated glycine aminoacyl tRNA synthetase (GARS) (Figure 3A). Two other interesting proteins were detected in this P1 versus control IP experiment, including AFG3L2, an ATP-dependent protease localized to the mitochondrial inner membrane. Curiously, the control serum immunoprecipitated KIFC1, a mitotic kinesin molecule. MS analysis of the P3 IP products showed a very strong IP of alanine aminoacyl tRNA synthetase (AARS), without other significant autoantigens (Figure 3C).

P2 and P4 sera were obtained from patients with scleroderma with known anti-topoisomerase I antibodies.^42−46^ MS analysis of the P2 serum immunoprecipitate showed the presence of both cytoplasmic topoisomerase I (TOP1) and mitochondrial topoisomerase 1 (TOP1MT), two highly homologous proteins (Figure 3B). Serum P4 immunoprecipitated the most complex set of autoantigens (Figure 3D). In addition to expected TOP1 and TOP1MT, three other proteins were immunoprecipitated, DLAT (dihydrolipoyllysine-residue acetyltransferase component of pyruvate dehydrogenase complex, mitochondrial), CENPC (centromere protein C), and PDHX (pyruvate dehydrogenase protein X component, mitochondrial) (Figure 3D).

The normalized intensities corresponding to specific autoantigen targets found were plotted for each IP sample (Figure 3). These data demonstrate the high degree of reproducibility of this method with a tight clustering of each protein within each IP group. The associated FDR adjusted *P*-value for each protein is shown in parentheses. Thus, the IP-to-MS workflow was able to detect both known autoantigens and novel autoantigens in a highly reproducible manner.

### Comparison of IP-to-MS to Other IP Methods

To further demonstrate the utility of the IP-to-MS workflow, we compared our IP-to-MS method to conventional IP and IP with immunoglobulins chemically cross-linked to protein A resin. In all three experiments, target/substrate proteins derived from K562 cell lysate were labeled with Cy3-NHS. Serum proteins (including immunoglobulins) bound to protein A resin were Cy5-labeled in situ. Four sera from either myositis or scleroderma patients with autoantibodies of known specificity were assessed. Included in this experiment were a negative control serum from a healthy/nondiseased patient and a positive control preparation of purified rabbit polyclonal antibodies targeting Hsp90.

**Figure 4:**
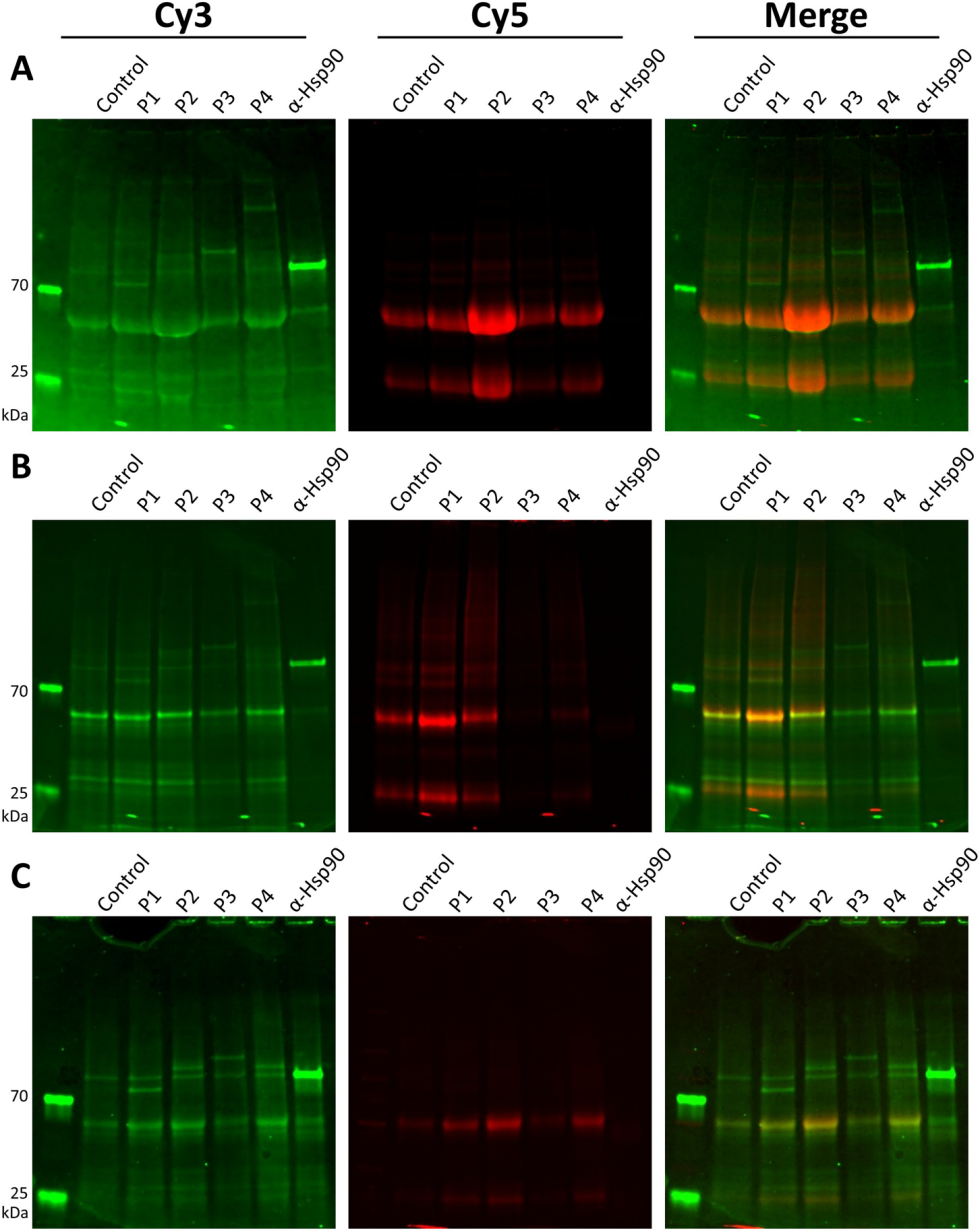
IP of a standard set of sera samples and anti-Hsp90 without ProMTag (A), without ProMTag and with cross-linking of antibodies to protein A resin (B), and with ProMTag (C). IPs were carried out with a set of sera or anti-Hsp90. For each set of IPs, the K562 lysate was labeled with Cy3-NHS and the antibodies were labeled with Cy5-NHS. (A) IPs were carried out on lysates that were not labeled with ProMTag and not cleaned-up with TCO resin. (B) IPs were carried out identically to those in (A) with the exception that the antibodies were cross-linked to the protein A resin prior to binding the Cy3-labeled K562 lysate. (C) IPs were carried out with ProMTagged K562 lysate. ProMTag was added to the K562 lysate immediately after addition of Cy3-NHS. The eluates from the protein A resin were added to TCO resin and incubated for 15 min to allow binding of ProMTagged target proteins. The TCO resin was then washed and cleaned-up target proteins were eluted from the TCO resin.

First, we performed conventional IPs without ProMTag or chemical cross-linking (Figure 4A). The Cy3-labeled target proteins are shown in green, while the Cy5-labeled immunoglobulins are shown in red. The negative control lane shows a set of Cy3-labeled proteins from the target cell lysate that were nonspecifically retained by the workflow. This same set of proteins was observed across all patient sera lanes, but not in the purified rabbit antibody lane, indicating that the presence of either bound immunoglobulins or other serum associated proteins aided in the nonspecific binding of target cell lysate. In addition to these so-called background bands, the patient sera precipitated additional distinct protein bands. The Cy5 fluorescence image also revealed a variable amount of immunoglobulin associated with each serum sample.

A commonly used method for separating immunoglobulins from antigens during IP is to chemically cross-link immunoglobulins to the protein A resin. Chemical cross-linking has certain limitations.^18,47−49^ If the extent of cross-linking is low, then immunoglobulin will contaminate the final IP product. Conversely, if the cross-linking is too aggressive, there is potential to block the antigen binding sites, reducing the efficacy of IP. Thus, typical IP experiments involving chemical cross-linking require optimization of the extent of cross-linking reagent. Here, we used the recommended conditions provided by the manufacturer, without optimization for each serum tested. Cy3 fluorescence demonstrated that cross-linking improved the resolution of the IP gels (Figure 4B). However, the background bands persisted. Furthermore, Cy5 fluorescence showed that, while the level of immunoglobulin contamination was reduced compared to the non-cross-linked samples, the variable degree of immunoglobulin removal indicated insufficient cross-linking in some samples.

Using double-labeled Cy3, ProMTagged cell lysate in the IP workflow showed that the level of background Cy3-labeled proteins was reduced relative to the Cy3-target proteins (Figure 4C, note the relative intensity of the target protein bands to the background bands seen in the control lane is stronger). This reduction in background signal was most likely due to the more stringent washing of the TCO resin afforded by the ProMTag covalent linkage, though it is important to point out that there was still some immunoglobulin breakthrough in the final IP product. Overall, this breakthrough appeared to be less significant than the immunoglobulin contamination observed in the cross-linking experiment (compare Figure 4B,C merged images).

Finally, to confirm the identity of the Cy3-target protein bands in these gels, the bands were excised from their respective gels and the proteins were rapidly digested with MT-Trypsin and analyzed by MS (Figure S3). Shown are the top ten proteins identified by MS for each of the 21 protein bands excised from the three IP gels where there was no treatment of the antibodies bound to the protein A resin or ProMTagging of the K562 lysate (Figure S3A); the antibodies were cross-linked to the protein A resin (Figure S3B); or, the K562 lysate was labeled with ProMTag (Figure S3C).

Sera labeled P1 and P3 derived from myositis patients and were known to contain antibodies against glycine tRNA synthetase and alanine tRNA synthetase, respectively.^50^ Bands 2, 9, and 16, which were cut from P1 lanes, were confirmed to contain glycine tRNA synthetase, while bands 4, 11, and 18, which were cut from P3 lanes, were confirmed to contain alanine tRNA synthetase, as expected (Figure S3). The P2 and P4 sera were obtained from patients with scleroderma and were known to contain anti-topoisomerase I antibodies.^42−46^ Bands 3, 6, 10, 13, 17, and 20, which were cut from the P2 and P4 lanes, were all confirmed to contain topoisomerase I as expected. These data clearly confirmed that the various IP methods isolated the expected proteins, but importantly the ProMTagged products appeared to be the most contaminant- and immunoglobulin-free.

### Comparison of IP-to-MS to Conventional IP, Double Immunodiffusion, and ELISA

Systemic sclerosis (SSc) encompasses a group of autoimmune diseases characterized by tissue fibrosis, vascular disease, and autoantibody production. The most prevalent SSc-related autoantibodies include anti-centromere, anti-topoisomerase I (ATA), and anti-RNA polymerase III. Importantly, these autoantibodies mark distinct subsets with varied clinical phenotypes and disease outcomes/prognoses. As an example of these phenotype–serotype associations, ILD is the most common cause of death in SSc patients and is more prevalent among patients positive for ATA, demonstrating that ATA is an indicator of SSc prognosis.^51^ Accurate autoantibody identification is therefore crucial for optimal clinical management.

Currently, IP is the gold standard method for identifying most SSc-specific autoantibodies, but the reliability of this assay in determining the presence of ATA is questionable given that topoisomerase I appears at the same band size as multiple other human proteins/target antigens. Ouchterlony DID represents an alternative method of detecting ATA in SSc patient sera, but there has been little research on its comparative accuracy and reliability relative to IP or other highly sensitive, but potentially less specific, commercial methods such as ELISA.

To address these questions, we compared the accuracy of conventional IP, ELISA, DID, and IP-to-MS in the detection of ATA in SSc patients. Sera derived from a group of SSc patients from the University of Pittsburgh longitudinal cohort previously classified as ATA positive (ATA+) by conventional IP, DID* (calf thymus extract used as substrate), or commercial ELISA were reassessed by custom ELISA and DID assays using recombinant human topoisomerase I as substrate antigen.

We assessed 82 serum samples through our custom ELISA. As shown in Table S2, 66/82 samples exceeded the threshold of 0.15 arbitrary units (AU), which was established based on the mean standardized antibody level (+2 SD) in a healthy control cohort. Based on the distribution of ELISA values across the full cohort, we further stratified patients into three groups: high titer (≥30th percentile rank of cohort), low titer (<30th percentile), and negative (<0.15 AU).

When assessed by our custom Ouchterlony DID assay, 63/82 samples yielded precipitin lines consistent with the presence of ATA (Table S2). Five of the 19 samples that were negative by our custom DID were low titer positive ATA by our ELISA. Of the 66 samples with ELISA evidence of ATA, 61 generated DID patterns indicative of ATA positivity, and 61/63 samples positive for ATA by DID were also positive by ELISA (Table S3). The overall concordance of ELISA and DID was 91%. Based on the assumption that DID represents the conventional “gold standard” for accurate detection of ATA, the relative sensitivity and specificity of our custom ELISA was 0.968 and 0.667, respectively. Because 18% (15/82) of the cohort previously classified as ATA+ by conventional IP was classified as ATA negative (ATA−) by DID, our results suggested that conventional IP alone is unreliable in accurately identifying ATA positivity.

To address this apparent discrepancy, we selected 13 SSc+ patient sera with discordant ATA assessments (ATA+ by conventional IP, DID*, or commercial ELISA, but ATA– by our custom DID) for analysis by IP-to-MS (Table 1, P1–P8, P10, P12, P13, P15, and P16; and Table S2). Six sera from SSc+ patients that were deemed ATA– by conventional IP or commercial ELISA were included as an SSc+ ATA– control group (Table 1, P9, P11, P14, and P17–P19). In addition, seven ATA+ sera (positive by conventional IP, as well as our custom ELISA and DID assays) were selected as reference samples (Table 1, P20–P26). Sera from six healthy individuals comprised the negative control group (Table 1, P27–P32).

**Table 1:**
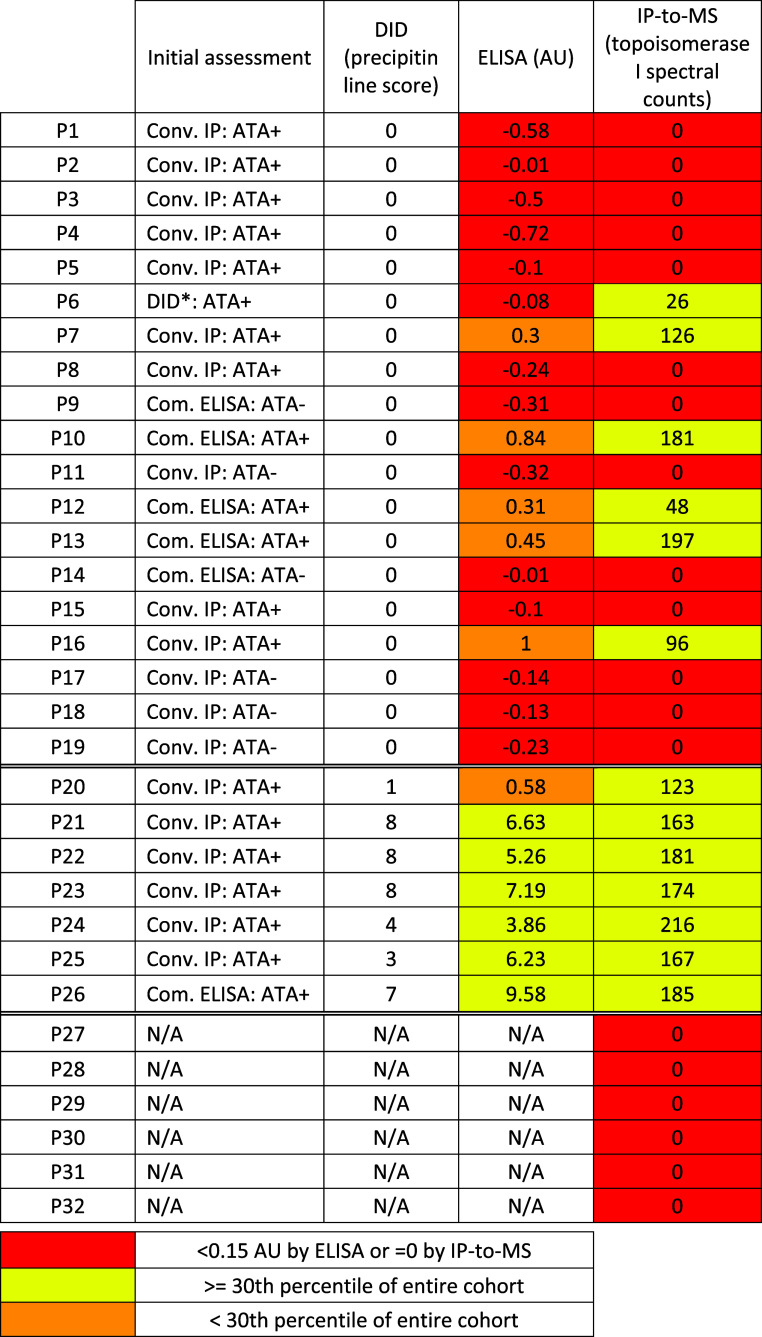
Patient Sera Selected for Analysis by IP-to-MS

**Figure 5:**
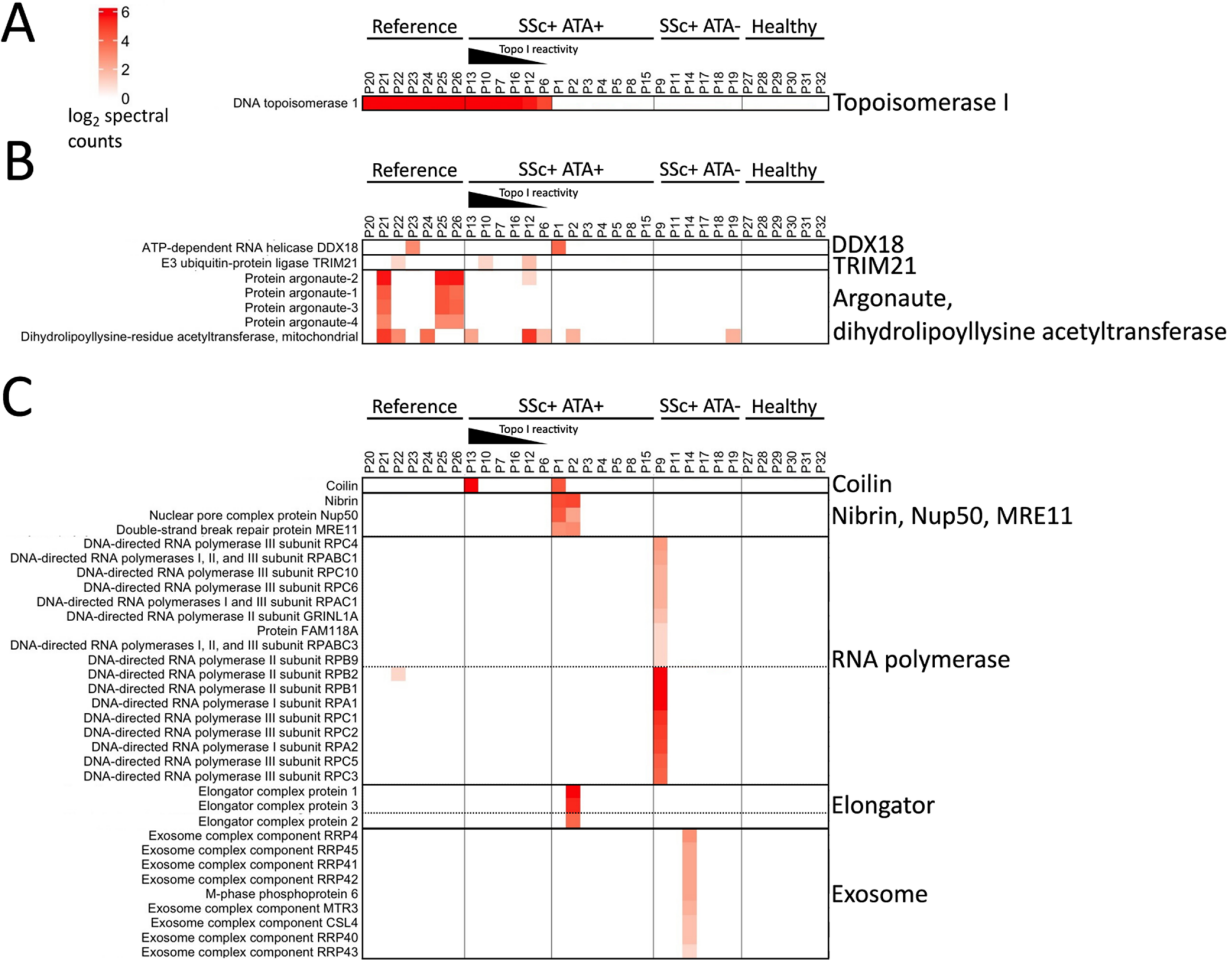
IP-to-MS of 32 patient sera against K562 lysate. IP-to-MS of 32 patient sera were carried out against a PerMTagged K562 lysate. These samples were broken into four groups. The first group of samples (P20–P26; reference) was prepared with patient sera which were ATA+ by conventional IP and by our custom ELISA and DID assays. The second set (P1–P8, P10, P12, P13, P15, and P16; SSc+ ATA+) was prepared with patient sera that had initially been classified as ATA+ by conventional IP, DID*, or commercial ELISA, but were ATA– by our custom DID assay. The third group of samples (P9, P11, P14, and P17–P19; SSc+ ATA−) was prepared with sera from SSc+ patients that were deemed ATA– by conventional IP or commercial ELISA. The fourth group of samples (P27–P32; healthy) was prepared with sera from five healthy individuals. MS data were filtered to remove immunoglobulin, keratin, and trypsin, and the data were displayed as a heatmap where the log_2_ spectral counts were plotted. Select areas of the heatmap with protein complexes of interest are shown (A–C). The full heatmap is shown in Figure S4.

With IP-to-MS, quantifiable topoisomerase I was detected in 13 samples (Table 1, Figure 5A) based on spectral counts of unique topoisomerase I peptides. All of the reference samples (7/7) were high titer. Six of the ATA– (by custom DID) samples (6/19) also had detectable topoisomerase I by IP-to-MS; five were strongly positive and one was weakly positive. Topoisomerase I was undetectable in 13 of the test samples (13/19), all of which were also ATA– by both ELISA and DID. Importantly, none of the healthy control sera contained topoisomerase I reactivity.

Overall, there was very good concordance between the four immunoassays among the reference samples, with the exception of P20 that demonstrated low titer positivity by ELISA, but high titer positivity based on IP-to-MS spectral counts. Among the test group, 5 samples demonstrating low titer positive ATA by ELISA yielded high-titer positive results by IP-to-MS. One sample (P6) was negative by ELISA and DID but tested positive by IP-to-MS. Collectively, these results indicate that IP-to-MS may be a more sensitive measure of anti-topoisomerase I antibodies than conventional IP, DID, or ELISA.

IP-to-MS provides a survey of the target cell lysate’s whole proteome. Figure S4 shows a heatmap of proteins captured by the IP-to-MS workflow for the 32 sera examined. As in Figure S2, there were nonspecific binding proteins detected in the reference, test, and healthy control sera. Topoisomerase I was not the only protein specifically precipitated by the reference and test sera. These other proteins included argonaute subunits, TRIM21, DDX18, and dihydrolipoyllysine acetyltransferase (Figure 5B), demonstrating that IP-to-MS can also serve as an autoantigen discovery tool. As further evidence of this diagnostic utility, the test sera that were topoisomerase I negative contained antibodies to novel antigens, including coilin, nibrin, MRE11, Nup50, elongator proteins, RNA polymerases, and exosome subunits (Figure 5C).

Taken together, these data show that IP-to-MS may outperform conventional IP, DID, and ELISA in identifying sera that contain ATA. Equally important, IP-to-MS has the capacity to identify additional, potentially novel/undiscovered autoantigens recognized by patient sera, allowing for further stratification of patients than is possible with conventional methods of autoantibody detection.

## Discussion

We present here a novel IP-to-MS method for detection of disease-associated autoantibodies that is founded on coupling ProMTag, a reversible protein tag that forms a rapid irreversible covalent bond to a bead-based matrix using click chemistry, to an unbiased pool of potential target proteins. ProMTagged protein lysate is coincubated with antibodies from patient sera bound to protein A resin to capture autoantigens. After washing away unbound proteins, the ProMTagged autoantigens are released under strong denaturing conditions along with the immunoglobulins that were bound to the protein A resin. The ProMTagged autoantigens are separated from the large excess of immunoglobulins by covalent coupling to click chemistry resin using the pairing of MT and TCO. Finally, the autoantigens are released from the ProMTag, digested with MT-Trypsin, and analyzed by MS.

We have previously shown that ProMTag serves as a universal protein tag which is capable of capturing proteins from a wide variety of sources.^24^ We have also previously shown that MT-Trypsin is able to rapidly digest proteins while being tethered to TCO resin, thus enabling the addition of relatively large amounts of trypsin that do not overly contaminate peptide samples. We show here that ProMTag is suitable for labeling native proteins and that the linkage is maintained over hours long incubation times. We also show that the IP-to-MS method is highly reproducible and faithfully captures known autoantigens from previously characterized patient sera.

A “real world” test of the IP-to-MS workflow of 37 samples consisting of 15 positive controls containing autoantibodies of known specificity, 5 negative controls, and 17 test samples (“unknowns”) derived from patients with CTD-ILD provided a wealth of information. All but one positive control yielded the expected autoantigen target, and all of the test samples yielded candidate autoantigen targets. Several of the test sera autoantigens matched those recognized by sera from positive control samples. Because the ProMTag was added under native conditions, many of the IPs contained protein complexes. Although our current IP-to-MS method does not distinguish between direct and indirect autoantibody binding, future development of this workflow will optimize conditions for mild complex disruption to identify the direct autoantigen targets.

Data from IP-to-MS studies will be useful for content-rich, molecular characterization of autoimmune diseases. The current state-of-the-art approach to autoimmune disease diagnosis includes a combination of clinical evaluation and targeted autoantibody testing but is limited to assessment of known autoantigens included in commercially available tests that are often ELISA-based. Seropositive samples based on ELISA assays are scored by magnitude of reactivity, while IP, cytometric, and immunodiffusion assays mostly yield binary positive or negative results that are difficult to quantify with precision.

The identification of new autoantigens traditionally requires in-depth bench-side analysis involving multistep, cumbersome methods such as immunoblotting followed by gel matching, band excision, and MS analysis. Novel autoantigen discovery has required sophisticated experimental techniques and research expertise. The IP-to-MS workflow bypasses many of the complex steps required for novel autoantigen discovery. The entire workflow takes ∼6 h to complete and is technologically simple, involving routine microcentrifuge spin-isolation handling. IP-to-MS also obviates the need for targeted autoantigen tests since the method is unbiased and can be targeted to any proteome source.

A key advantage of the IP-to-MS method is that it produces quantitative data that can be entered into public data repositories for interstudy comparisons. This will allow for quantitative longitudinal analysis to follow the progress of a disease and evaluate the efficacy of a treatment regime. It also enables large-scale, study-to-study comparisons for in-depth statistical analysis. The data analysis presented here only required commonly used analytical tools. Collecting larger data sets will allow for machine-learning and AI approaches to assess these quantitative data.

Finally, the IP-to-MS workflow is organism, disease, and condition agnostic, as it can be applied to any IP experiment and is not limited by source of antibody or type of cell/tissue extract/substrate. Of particular interest, this method will have important applications in other disciplines such as cancer immunology, as all cancers elicit an immune response.^52,53^ Harnessing the IP-to-MS method to identify patient-specific cancer antigens will provide an additional level of molecular characterization of a tumor, as well as a measure of cancer load, treatment risk (e.g., immune checkpoint inhibitor-induced adverse events), treatment response, and relapse, effectively establishing the foundation for development of personalized treatment regimes.

## Data Availability

The MS proteomics data have been deposited to the ProteomeXchange Consortium via the PRIDE (https://www.ebi.ac.uk/pride/) partner repository with the data set identifier PXD056617. These data sets are associated with the following figures: IP1–37; Figures 2 and S2 Minden_051822; Figure 3 ScIP1–32_OctNov2023; Figure 5, Figure S4 Gel1–26_Jan2024; Figure S3.

## Supporting Information Available

Gel-based validation of the ProMTag IP workflow (Figure S1). Full heatmap for IP-to-MS of 37 patient sera against K562 lysate, from which Figure 2 is derived (Figure S2). MS characterization of bands cut from gels shown in Figure 4 (Figure S3). Full heatmap for IP-to-MS of 32 patient sera against K562 lysate, from which Figure 5 is derived (Figure S4). Unedited gel images for gels shown in Figure 4, Figures S1 and S3 (Figures S5 and S6). Clinical diagnoses of patients shown in Figures 2 and S2 (Table S1). SSc patient sera analysis by DID and ELISA (Table S2). Contingency table analysis of DID vs ELISA results for ATA positivity (Table S3) (PDF)
Filtered data used to generate Figure 2/Figure S2 heatmap (Table S4) (XLSX)
Filtered data used to generate Figure 5/S4 heatmap (Table S5) (XLSX)
MS data shown in Figure 3 (Table S6) (XLSX)
