# Supplementary material for: IP-to-MS: An Unbiased Workflow for Antigen Profiling
Source: J Proteome Res. 2025 Jan 15;24(2):795–812. doi: 10.1021/acs.jproteome.4c00837 (PMC11812086; doi:10.1021/acs.jproteome.4c00837)
Supplement: Supplementary file 1 — pr4c00837_si_001.pdf [file pr4c00837_si_001.pdf]

IP-to-MS: an unbiased workflow for antigen profiling

Supporting Information

Stephanie Biedka[1], Svitlana Yablonska[1], Xi Peng[2,3], Duah Alkam[4], Mara Hartoyo[5], Hannah VanEvery[5], Daniel J. Kass[6], Stephanie D. Byrum[4,7,8], Kunhong Xiao[2,3], Yingze Zhang[6], Robyn T. Domsic[9], Robert Lafyatis[9], Dana P. Ascherman[9,*], Jonathan S. Minden[1,*]

[1]Impact Proteomics, LLC., Pittsburgh, PA 15206, USA

[2]Center for Proteomics & Artificial Intelligence, Allegheny Health Network Cancer Institute, Pittsburgh, PA 15205, USA

[3]Center for Clinical Mass Spectrometry, Allegheny Health Network Cancer Institute, Pittsburgh, PA 15205, USA

[4]Department of Biochemistry and Molecular Biology, University of Arkansas for Medical Sciences, Little Rock, AR 72205, USA

[5]University of Pittsburgh School of Medicine, Pittsburgh, PA 15261, USA

[6]Division of Pulmonary and Critical Care Medicine, Department of Medicine, University of Pittsburgh School of Medicine, Pittsburgh, PA 15261, USA

[7]Arkansas Children's Research Institute, Little Rock, AR 72202, USA

[8]Department of Biomedical Informatics, University of Arkansas for Medical Sciences, Little Rock, AR 72205, USA

[9]Division of Rheumatology and Clinical Immunology, Department of Medicine, University of Pittsburgh School of Medicine, Pittsburgh, PA 15261, USA

*Corresponding authors: Dana Ascherman Email: dascher@pitt.edu Phone: (412) 383-8123, Jonathan Minden Email: jonathan.minden@impactproteomics.com Phone: (412) 268-2669

Table of Contents:

Figure S1. Gel-based validation of the ProMTag IP workflow.
Figure S2. IP-to-MS of 37 patient sera against K562 lysate heatmap.
Figure S3. MS characterization of bands cut from gels shown in Figure 4.
Figure S4. IP-to-MS of 32 patient sera against K562 lysate heatmap.
Figure S5. Unedited gel images for gels shown in Figure S1.
Figure S6. Uncropped gel images for gels shown in Figure 4 and Figure S3.
Table S1. Clinical diagnoses of patients in Figure 2 and Figure S2.
Table S2. SSc patient sera analysis by DID and ELISA.
Table S3. Contingency table analysis of DID vs. ELISA results for ATA positivity.
Table S4. Filtered data used to generate Figure 2/Figure S2 heatmap. Separate Excel file.
Table S5. Filtered data used to generate Figure 5/S4 heatmap. Separate Excel file.
Table S6. MS data shown in Figure 3. Separate Excel file.

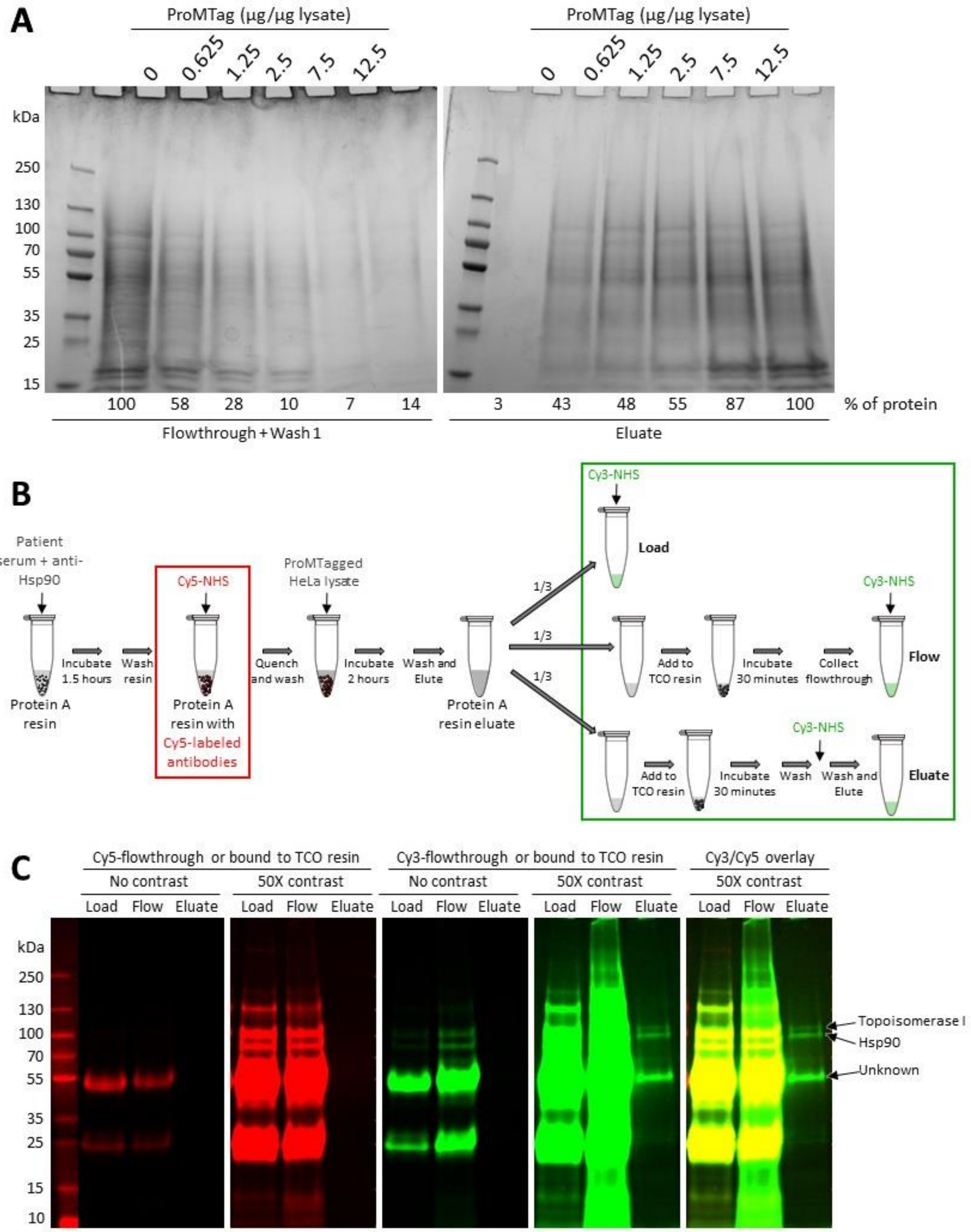

Figure S1. Gel-based validation of the ProMTag IP workflow. **(A)** K562 lysate was labeled with 7.5-12.5 µg ProMTag per microgram of protein lysate. The reaction was quenched, and the labeled lysate was incubated at 4 °C for 2 hours, then the lysate was bound to TCO resin. Coupling of ProMTag to protein is done at pH 8.0, while this linkage is reversed by brief exposure to pH 3.0. All incubations were carried out at the same temperature and for the same duration as in the IP workflow. The flowthrough and first wash fraction were combined and run on a SDS-PAGE gel, and the TCO resin eluate was run on a separate SDS-

PAGE gel. Both gels were stained with Coomassie Brilliant Blue R-250. Quantification of the SDS-PAGE gels was done with Image J, with the blank region between the third and fourth lanes used for background subtraction. The percent of protein remaining in the flowthrough + wash 1 or the percent of protein in the eluate was quantified according to the pixel intensity of the gel images using ImageJ. The protein amount was calculated as a percentage of the "0 ProMTag" control for the "Flowthrough + Wash 1" gel or as a percentage of the 12.5 μg ProMTag per μg lysate sample for the "Eluate" gel. **(B,C)** Separation of immunoglobulin from target proteins bound to TCO resin via ProMTag. Anti-Hsp90 antibody and a scleroderma patient serum standard known to possess anti-topoisomerase I antibodies were exposed to protein A resin. The immunoglobulins and other serum proteins that bound to the protein A resin were labeled in situ with CyDye Cy5-NHS. A ProMTagged HeLa cell lysate was then added to the protein A resin. After IP, the eluate from the protein A resin was collected and split in thirds. One-third was labeled with Cy3-NHS and retained as a load fraction (Load). One-third was loaded onto TCO resin, and the material that flowed through the TCO resin was retained as the flowthrough fraction and labeled with Cy3-NHS (Flow). The remaining one-third was also loaded onto TCO resin, but after a 30-minute incubation to bind the ProMTagged proteins to the resin, the TCO resin was washed extensively to remove unbound proteins, and the captured ProMTagged proteins were labeled with Cy3-NHS. The captured proteins were then released under acidic conditions (Eluate). These fractions were analyzed by SDS-PAGE and fluorescence imaging. Imaging for Cy5 fluorescence showed that the load and flowthrough fractions contained Cy5-labeled heavy and light immunoglobulin chains, while the eluate fraction had no detectable immunoglobulin when displayed with no contrast enhancement-scaling between the dimmest and brightest pixel value. Increasing the contrast fifty-fold revealed the presence of additional Cy5-labeled non-immunoglobulin proteins that bound to the protein A resin, while still showing no immunoglobulin in the eluate lane. Imaging the gel for Cy3 fluorescence also showed that the load and flowthrough fractions contained large amounts of immunoglobulin heavy and light chains. However, although the Cy3-eluate lane appeared blank when the image was displayed without contrast enhancement, increasing the image contrast 50-fold clearly demonstrated the immunoprecipitation of Hsp90 and topoisomerase I, plus an unidentified protein band.

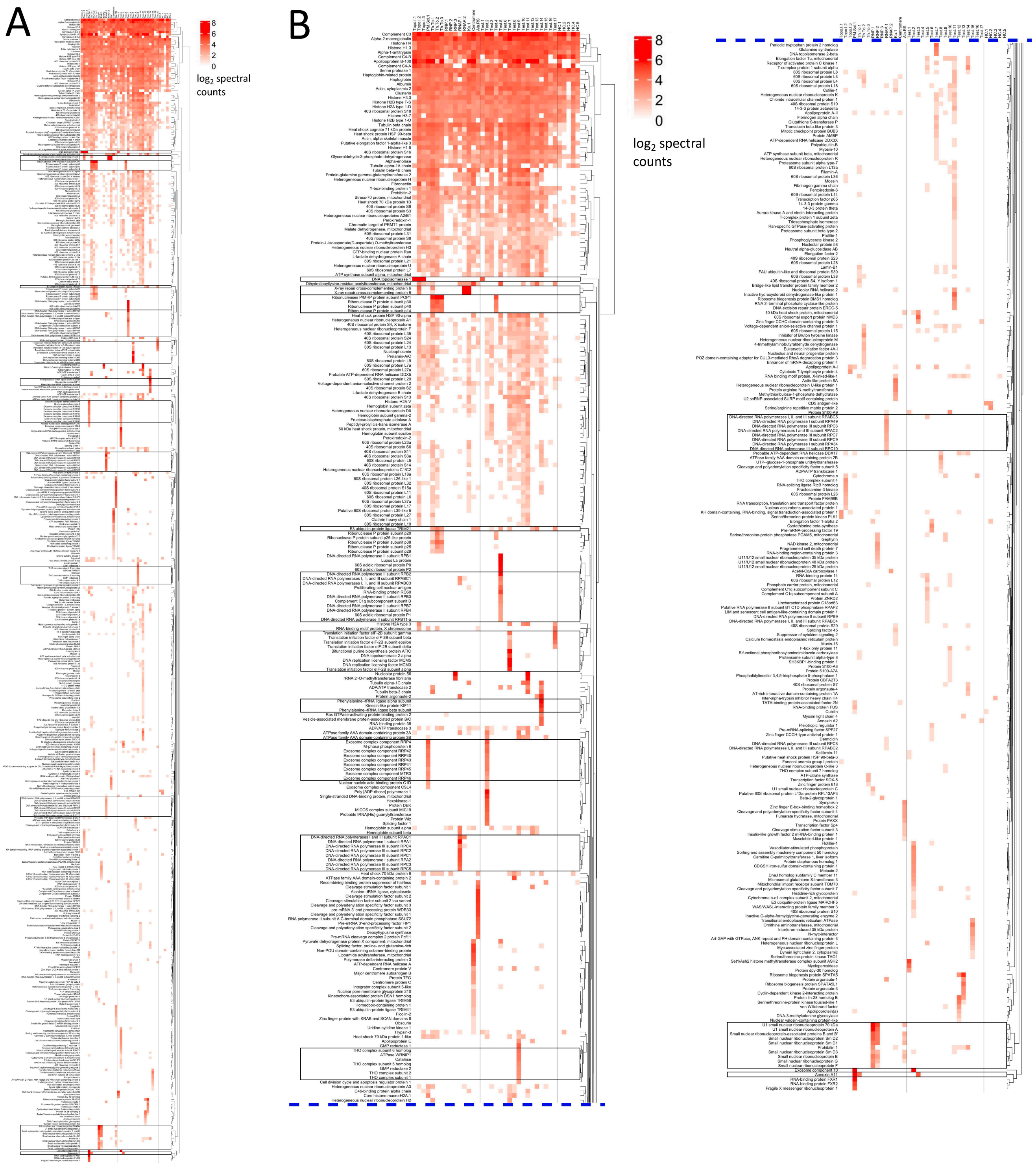

Figure S2. IP-to-MS of 37 patient sera against K562 lysate heatmap. Full heatmap from which data shown in Figure 2 was taken. Boxes indicate regions of interest shown in Figure 2. Both **(A)** and **(B)** show the full heatmap. The heatmap in **(A)** was cropped in half at the dotted blue line **(B)**.

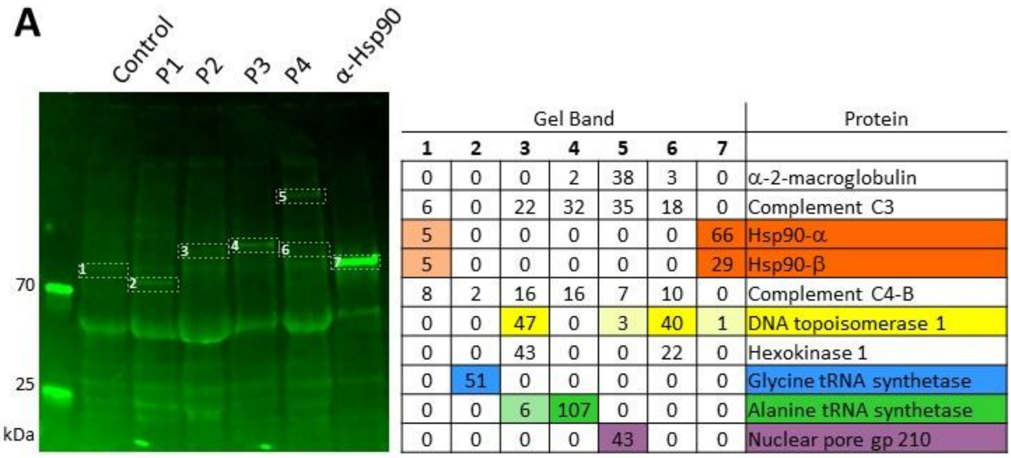

Gel Band							Protein
**1**	**2**	**3**	**4**	**5**	**6**	**7**	
0	0	0	2	38	3	0	α-2-macroglobulin
6	0	22	32	35	18	0	Complement C3
5	0	0	0	0	0	66	Hsp90-α
5	0	0	0	0	0	29	Hsp90-β
8	2	16	16	7	10	0	Complement C4-B
0	0	47	0	3	40	1	DNA topoisomerase 1
0	0	43	0	0	22	0	Hexokinase 1
0	51	0	0	0	0	0	Glycine tRNA synthetase
0	0	6	107	0	0	0	Alanine tRNA synthetase
0	0	0	0	43	0	0	Nuclear pore gp 210

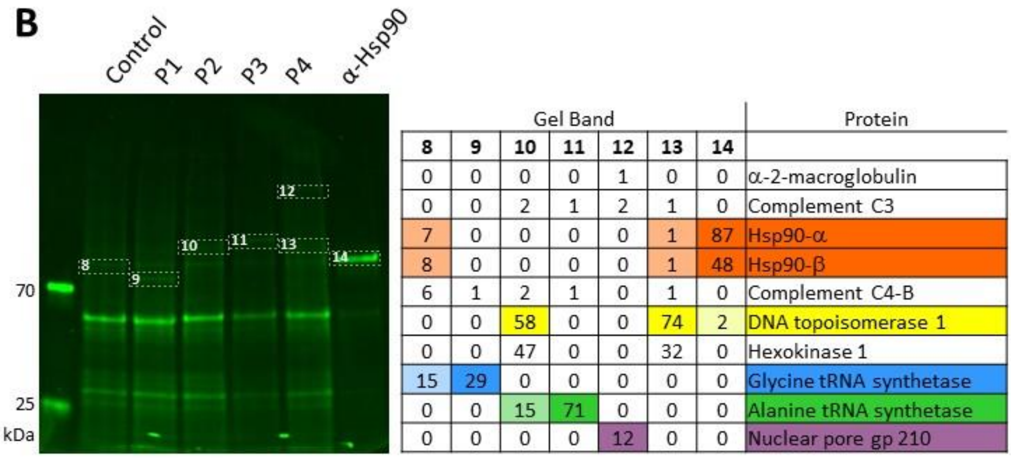

Gel Band							Protein
**8**	**9**	**10**	**11**	**12**	**13**	**14**	
0	0	0	0	1	0	0	α-2-macroglobulin
0	0	2	1	2	1	0	Complement C3
7	0	0	0	0	1	87	Hsp90-α
8	0	0	0	0	1	48	Hsp90-β
6	1	2	1	0	1	0	Complement C4-B
0	0	58	0	0	74	2	DNA topoisomerase 1
0	0	47	0	0	32	0	Hexokinase 1
15	29	0	0	0	0	0	Glycine tRNA synthetase
0	0	15	71	0	0	0	Alanine tRNA synthetase
0	0	0	0	12	0	0	Nuclear pore gp 210

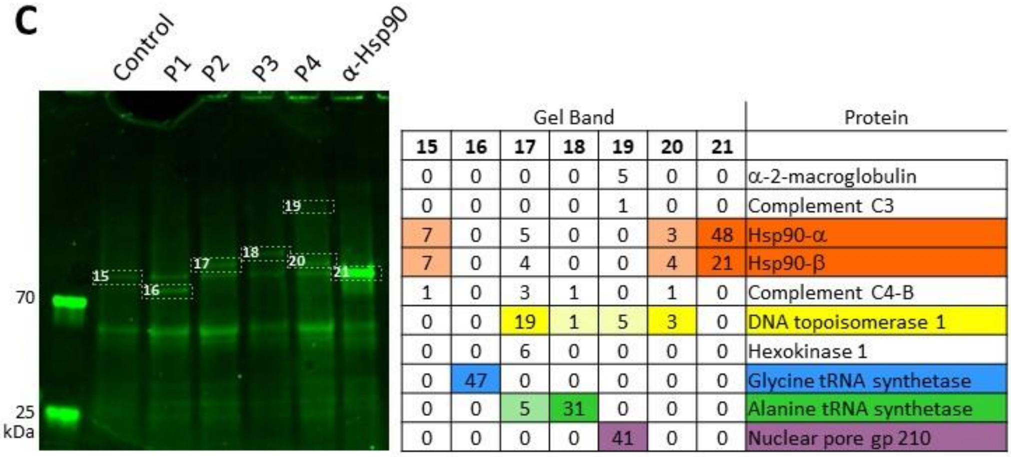

Gel Band							Protein
**15**	**16**	**17**	**18**	**19**	**20**	**21**	
0	0	0	0	5	0	0	α-2-macroglobulin
0	0	0	0	1	0	0	Complement C3
7	0	5	0	0	3	48	Hsp90-α
7	0	4	0	0	4	21	Hsp90-β
1	0	3	1	0	1	0	Complement C4-B
0	0	19	1	5	3	0	DNA topoisomerase 1
0	0	6	0	0	0	0	Hexokinase 1
0	47	0	0	0	0	0	Glycine tRNA synthetase
0	0	5	31	0	0	0	Alanine tRNA synthetase
0	0	0	0	41	0	0	Nuclear pore gp 210

Figure S3. MS characterization of bands cut from gels shown in Figure 4. Gels are as follows: **(A)** no treatment of the antibodies bound to the protein A resin or ProMTagging of the K562 lysate, **(B)** antibodies were crosslinked to the protein A resin, **(C)** the K562 lysate was labeled with ProMTag. The indicated bands were cut from the gels and prepared for MS via in-gel digestion with MT-Trypsin. The top ten proteins identified by MS in each of the bands are shown in the tables to the right of each gel. Light colored cells (i.e. the light green cell for band 3) indicate that the protein is predicted to be contamination due to gel handling or crossover from the neighboring band.

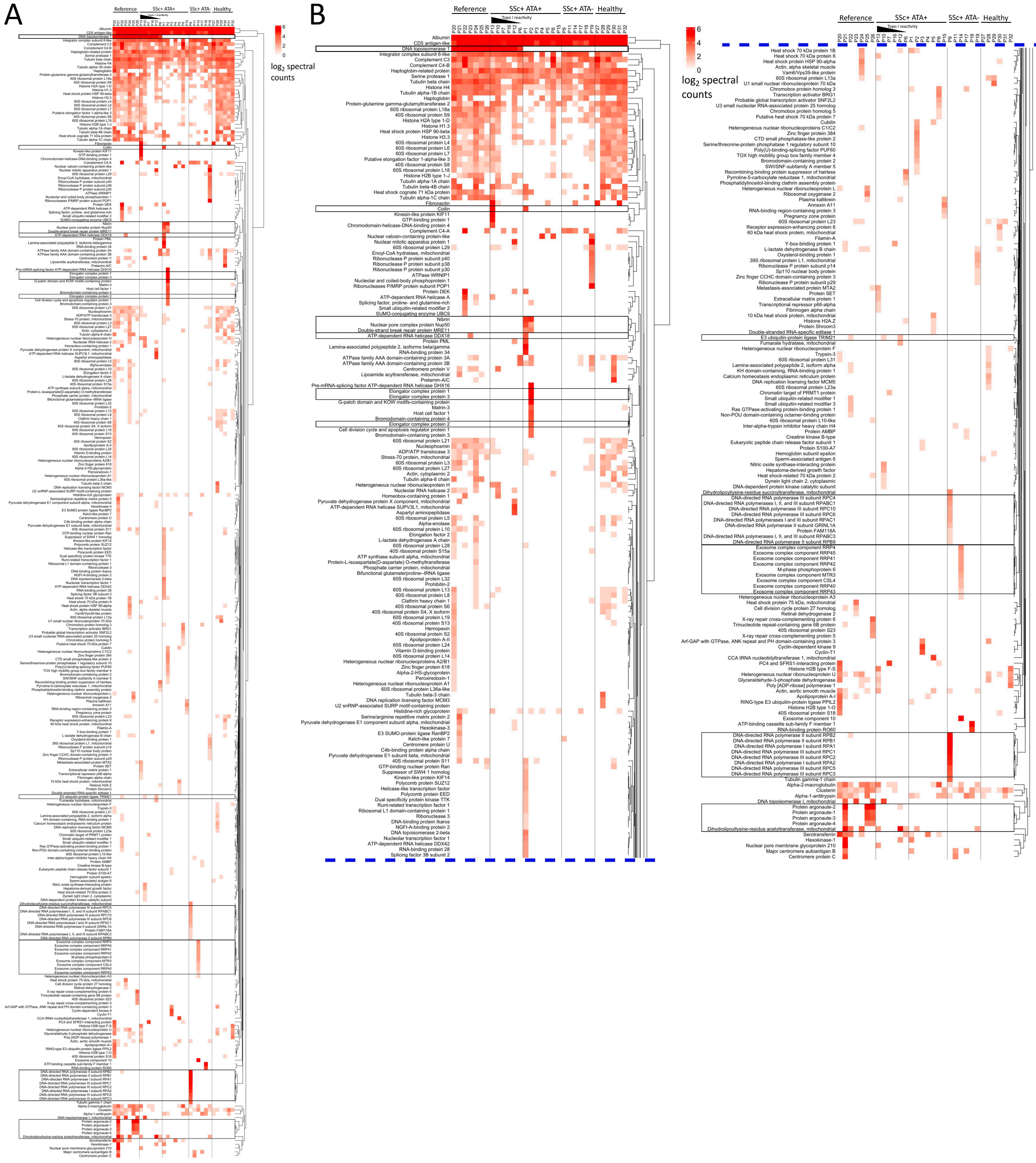

Figure S4. IP-to-MS of 32 patient sera against K562 lysate heatmap. Full heatmap from which data shown in Figure 5 was taken. Boxes indicate regions of interest shown in Figure 5. Both **(A)** and **(B)** show the full heatmap. The heatmap in **(A)** was cropped in half at the dotted blue line **(B)**.

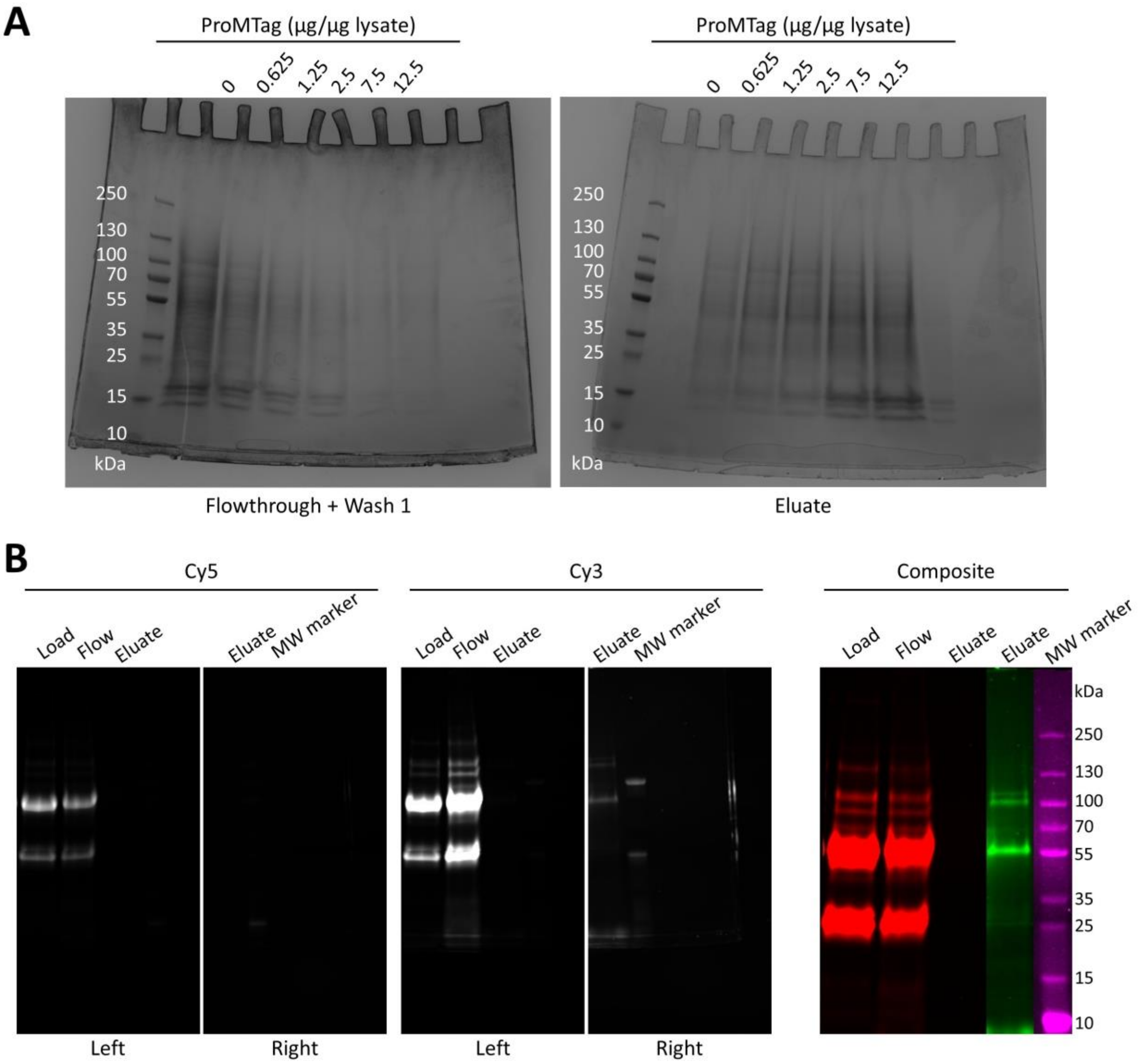

Figure S5. Unedited gel images for gels shown in Figure S1. **(A)** Unedited gels shown in Figure S1A. **(B)** Unedited gels shown in Figure S1C.

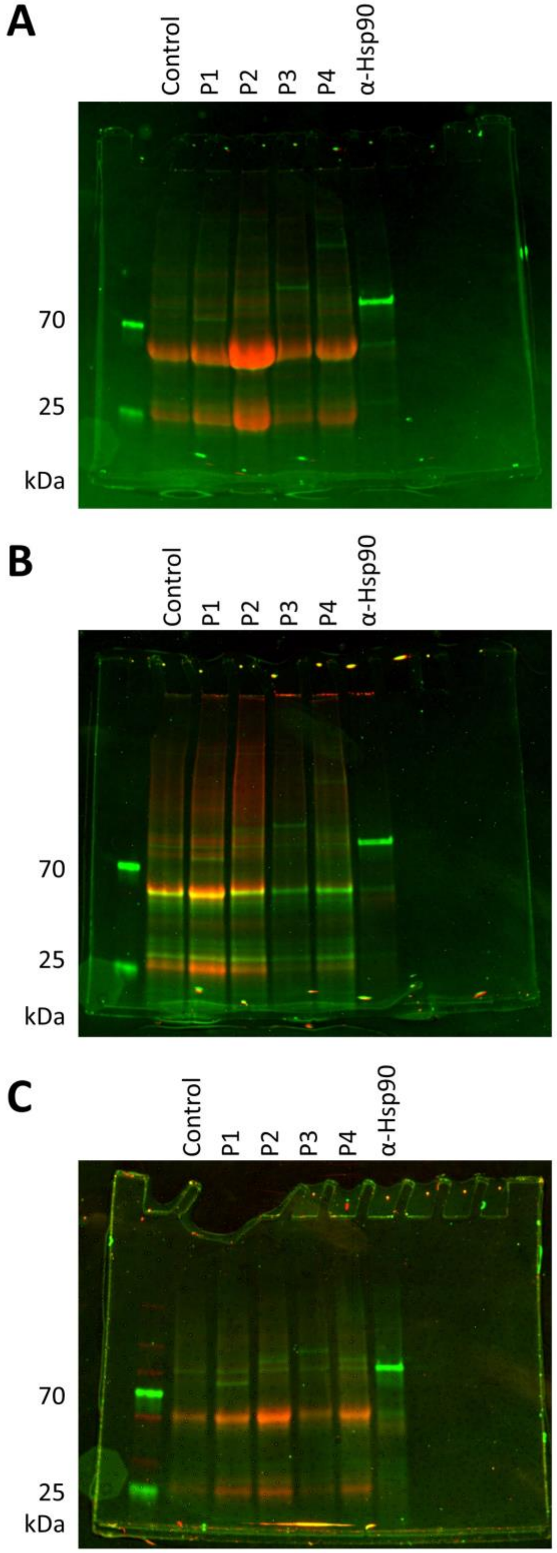

Figure S6. Uncropped gel images for gels shown in Figure 4 and Figure S3.

Table S1. Clinical diagnoses of patients in Figure 2 and Figure S2.

Figure 2 Group	Patient ID Figure 2	Clinical Diagnosis
Previously characterized patient sera (known autoantigens)	Topo.I.1	lcSSc
	Topo.I.2	dcSSc
	Topo.I.3	dcSSc
	PM.Scl.1	lcSSc/dermatomyositis overlap
	Th.To.1	lcSSc
	Th.To.2	lcSSc
	Th.To.3	lcSSc
	RNP.1	lcSSc
	RNP.2	lcSSc
	RNP.3	lcSSc
	RNAP.1	dcSSc
	RNAP.2	dcSSc
	Ku.1	dcSSc
	Centromere	lcSSc
	Ala.RS	dcSSc
Uncharacterized patient sera	Test.1	lcSSc
	Test.2	lcSSc
	Test.3	Unclassified: IgG4-related disease versus sine SSc skin disease
	Test.4	lcSSc
	Test.5	Systemic lupus erythematosus/Sjögren's syndrome
	Test.6	dcSSc
	Test.7	lcSSc
	Test.8	lcSSc
	Test.9	dcSSc
	Test.10	sine SSc
	Test.11	lcSSc
	Test.12	Unclassified
	Test.13	lcSSc
	Test.14	sine SSc
	Test.15	Unclassified, anti-synthetase syndrome/dermatomyositis
	Test.16	Unclassified
	Test.17	dcSSc
Healthy control sera	HC.1	Healthy control
	HC.2	Healthy control
	HC.3	Healthy control
	HC.4	Healthy control
	HC.5	Healthy control

dcSSc=diffuse cutaenous systemic sclerosis: lcSSc=limited cutaneous systemic sclerosis

Table S2. SSc patient sera analysis by DID and ELISA.

Patient	DID	ELISA (AU)	Selected test cases for IP-to-MS
1	Positive	3.87	
2	Positive	4.50	
3	Positive	2.98	
4	Negative	-0.58	P1
5	Negative	-0.01	P2
6	Positive	0.58	P20
7	Positive	6.63	P21
8	Positive	5.26	P22
9	Negative	-0.50	P3
10	Positive	6.21	
11	Positive	7.19	P23
12	Positive	3.86	P24
13	Positive	4.52	
14	Positive	6.23	P25
15	Positive	3.60	
16	Negative	-0.72	P4
17	Positive	1.04	
18	Positive	7.24	
19	Positive	2.30	
20	Positive	4.26	
21	Negative	-0.10	P5
22	Positive	0.70	
23	Positive	3.36	
24	Positive	1.51	
25	Negative	-0.08	P6
26	Negative	0.30	P7
27	Positive	2.23	
28	Positive	2.29	
29	Positive	0.58	
30	Positive	2.94	
31	Positive	0.23	
32	Positive	2.91	
33	Positive	2.73	
34	Positive	5.93	

35	Negative	-0.24	P8
36	Negative	-0.31	P9
37	Positive	0.52	
38	Positive	7.59	
39	Positive	10.14	
40	Positive	7.40	
41	Positive	1.43	
42	Positive	5.67	
43	Negative	0.84	P10
44	Positive	5.40	
45	Negative	-0.32	P11
46	Positive	2.51	
47	Negative	0.31	P12
48	Positive	0.14	
49	Negative	0.45	P13
50	Positive	3.87	
51	Positive	2.02	
52	Positive	2.75	
53	Positive	0.14	
54	Positive	4.88	
55	Positive	4.55	
56	Positive	6.12	
57	Negative	-0.01	P14
58	Positive	6.18	
59	Positive	4.29	
60	Positive	3.45	
61	Positive	6.12	
62	Positive	1.46	
63	Negative	-0.10	P15
64	Positive	4.84	
65	Positive	4.70	
66	Positive	0.39	
67	Positive	5.53	
68	Positive	6.29	
69	Positive	2.08	
70	Negative	1.00	P16
71	Positive	0.72	
72	Positive	2.08	
73	Negative	-0.14	P17

74	Negative	-0.13	P18
75	Negative	-0.23	P19
76	Positive	9.58	P26
77	Positive	8.62	
78	Positive	7.17	
79	Positive	2.03	
80	Positive	8.04	
81	Positive	2.60	
82	Positive	13.23	

Rows highlighted in red indicate patient samples that were selected to be analyzed by IP-to-MS as discordant conventional IP+ and DID-.

Rows highlighted in yellow indicate patient samples that were selected for IP-to-MS analysis as reference samples because they were ATA+ by conventional IP, DID, and ELISA.

Table S3. Contingency table analysis of DID vs. ELISA results for ATA positivity.

	DID+	DID-
ELISA+	61	5
ELISA-	2	10
